# Small Extracellular Vesicles from Neural Cells: Physiological and Pathological Roles, and Potential in Neurodegenerative Therapy

**DOI:** 10.1002/adhm.202504608

**Published:** 2026-04-06

**Authors:** Muhammad Waqas Salim, Wei Zhang, Lyndsey Collins‐Praino, Yuling Wang, Andrew Care

**Affiliations:** ^1^ School of Natural Sciences Macquarie University Sydney New South Wales Australia; ^2^ School of Pharmacy and Biomedical Science Adelaide University Adelaide South Australia Australia; ^3^ School of Life Sciences University of Technology Sydney Sydney New South Wales Australia

**Keywords:** Alzheimer's disease, neurodegenerative diseases, Parkinson's disease, small extracellular vesicles (sEVs), sEV engineering

## Abstract

Small extracellular vesicles (sEVs) have emerged as central mediators of intercellular communication in the central nervous system (CNS) and are increasingly recognized for their dual roles in the pathogenesis and treatment of neurodegenerative diseases (NDDs). In disease contexts, sEVs facilitate the intercellular dissemination of pathogenic proteins and nucleic acids, thereby contributing to the propagation of Alzheimer's disease (AD) and Parkinson's disease (PD) pathology. Conversely, their intrinsic biocompatibility, capacity to traverse brain barriers, and inherent organotropic properties position sEVs as highly promising nanocarriers for CNS drug delivery. While mesenchymal stem cell–derived sEVs have been widely investigated in preclinical NDD models, accumulating evidence suggests that sEVs derived from neural cells, including neural stem cells, neurons, astrocytes, microglia, oligodendrocytes, and brain endothelial cells may offer superior brain targeting, disease relevance, and functional efficacy. This review provides a comprehensive and critical analysis of current knowledge on neural cell–derived sEVs, encompassing their physiological roles in brain homeostasis, their involvement in AD and PD pathogenesis, and their emerging therapeutic applications. We discuss cell‐type–specific sEV cargo profiles, mechanisms underlying blood–brain and blood–cerebrospinal fluid barrier traversal, and recent advances in endogenous and exogenous engineering strategies that enhance cargo loading, targeting precision, and therapeutic performance. Importantly, we address key translational challenges that currently limit clinical implementation. By integrating mechanistic insights with therapeutic and engineering perspectives, this review highlights neural cell–derived sEVs as a biologically informed and versatile platform, underscoring their potential to advance next‐generation neuro‐nanomedicine for NDDs.

## Graphical abstract

Neural cell–derived small extracellular vesicles (sEVs) are emerging as pivotal mediators in neurodegenerative diseases, exerting both pathogenic and therapeutic functions. This review synthesizes current evidence on how sEVs from distinct neural cell types regulate neurodegeneration, neuroprotection, biomarker discovery, and targeted drug delivery, highlighting their organotropism, bioengineering potential, and translational promise in Alzheimer's and Parkinson's disease.

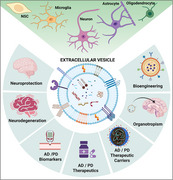

**Abbreviations**
AD: Alzheimer's disease
Aβ: amyloid‐β peptide
APP: amyloid precursor protein
EVs: extracellular vesicles
ESCRT: Endosomal Sorting Complex Required for Transport
MVs: microvesicles
MVB: multivesicular body
NDDs: neurodegenerative diseases
NFTs: neurofibrillary tangles
PD: Parkinson's disease
sEVs: small extracellular vesicles.

## 1 Introduction

The central nervous system (CNS) is an intricate network of neurons and glial cells. Neurons serve as the primary signaling units, forming complex electrical networks, while glial cells, including microglia, oligodendrocytes, and astrocytes, interact with neurons and one another to support and maintain CNS structure and function. Glial cells play essential roles in synaptogenesis, modulation of synaptic activity, metabolic support, and maintaining homeostasis [1, 2]. Unlike most other cell types, neural cells, particularly neurons, exhibit limited regenerative capacity. As a result, progressive neuronal damage can lead to the development of neurodegenerative diseases (NDDs), such as Alzheimer's disease (AD) and Parkinson's disease (PD). The preservation of nervous system integrity relies heavily on tightly regulated intercellular communication; any disruption to this communication can precipitate pathological states [1, 3, 4]. Neural cells communicate through diverse mechanisms including direct cell‐to‐cell contact, paracrine communication via the secretion of molecules in synapses, and vesicular interactions mediated by extracellular vesicles (EVs). EVs are recognized as pivotal conduits for cell‐to‐cell communication, particularly valued for their effectiveness over long distances [5].

**FIGURE 1:**
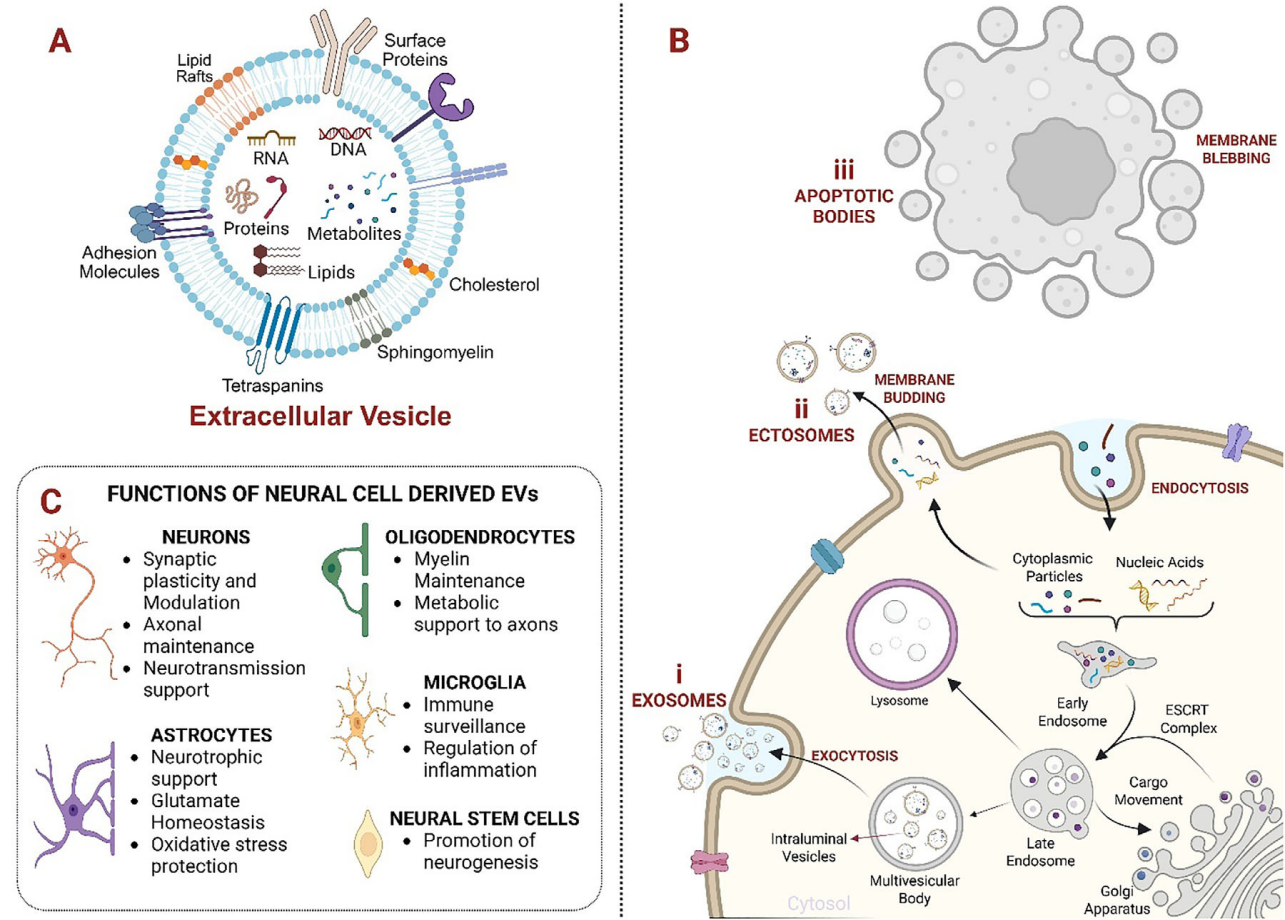
Composition, Biogenesis, and functions of extracellular vesicles (EVs). (A) Composition of EV showing diverse biomolecules, including lipids, proteins, nucleic acids, and metabolites (B) Biogenesis of EVs: (i) Exosomes are generated through the endosomal pathway, where multivesicular bodies fuse with the plasma membrane to release intraluminal vesicles. (ii) Ectosomes are formed by direct outward budding of the plasma membrane. (iii) Apoptotic bodies arise during membrane blebbing of dying cells. (C) Neural cell–derived EVs play their role in key physiological functions, including synaptic modulation, axonal maintenance, and neurotransmission by neuron‐derived EVs [10], neurotrophic, metabolic, and antioxidant support by astrocyte‐derived EVs [11], myelin maintenance and metabolic support by oligodendrocyte‐derived EVs [12] immune regulation and inflammation control by microglia‐derived EVs [13], and promotion of neurogenesis by neural stem cell‐derived EVs [14]. This image was created using Biorender.com.

EVs are lipid bilayer‐enclosed vesicles ranging from nano‐ to micro‐scale in size, released by nearly all cell types in the body [6]. These non‐replicating structures carry diverse biomolecules from their cells of origin, including proteins, nucleic acids, lipids, and metabolites, both on their surface and within their lumen (Figure 1A) [6, 7]. By transferring such molecular cargo, EVs facilitate a broad spectrum of biological processes.

EVs are formed through three principal biogenesis pathways: the endosomal route, direct plasma membrane budding, and apoptotic disassembly [8]. In the endosomal pathway, biogenesis begins with endocytosis, forming early endosomes that mature into multivesicular bodies (MVBs). Intraluminal vesicles (ILVs) are generated by inward budding of the endosomal membrane, with cargo sorted through ESCRT‐dependent or ESCRT‐independent mechanisms. MVBs either fuse with lysosomes for cargo degradation or with the plasma membrane to release ILVs as exosomes (Figure 1B‐i). The Golgi apparatus contributes to this process by supplying membrane components and facilitating molecular sorting via the trans‐Golgi network [8, 9]. Alternatively, EVs known as “ectosomes” are generated by outward budding of the plasma membrane (Figure 1B‐ii). A third type, apoptotic bodies, arise during programmed cell death through membrane blebbing and typically contain cellular organelles and nuclear fragments (Figure 1B‐iii) [8, 9].

Together, these pathways give rise to heterogeneous EV populations that reflect the physiological or pathological status of the parent cells. The “Minimal Information for Studies of Extracellular Vesicles (MISEV)” guidelines recommend that EV classification should prioritize size, unless the biogenesis origin is definitively known, a task that remains technically challenging [7]. EVs are broadly categorized as small EVs (sEVs, <200 nm) and large EVs (LEVs, >200 nm). Exosomes, derived via the endosomal route, are typically <200 nm, while ectosomes (sometimes referred to as microvesicles) range from 30 nm to several microns and originate from plasma membrane budding [7]. The term “microvesicles (MVs)” is discouraged by MISEV guidelines due to potential ambiguity, as ectosomes may also fall within the nanometer range [7].

All major neural cell types have been shown to release sEVs [15]. These vesicles, distinguished by their unique surface markers and cargo profiles, participate in several physiological processes essential for brain homeostasis. These include intercellular communication, cellular maintenance, removal of unnecessary intracellular components, and clearance of toxic substances [16]. The key functions of cell type–specific sEVs are summarized in Figure 1C, the details of which are given in the following sections. Given their diverse roles and abundance in biofluids, such as blood and cerebrospinal fluid (CSF) [17], sEVs are increasingly being explored as diagnostic and prognostic biomarkers of brain health. Conversely, emerging evidence also implicates sEVs in the pathogenesis and progression of neurodegenerative diseases. In particular, sEVs facilitate the intercellular transmission of pathological proteins, such as amyloid‐β and tau in AD, and alpha‐synuclein in PD, thereby contributing to disease spread [15]. This has led to growing interest in targeting sEVs therapeutically. For example, inhibition of sEV synthesis has been shown to attenuate the spread of pathological proteins in neurodegeneration models [18].

Owing to their lipid membranes, sEVs structurally resemble liposomes, making them well‐suited for encapsulating and delivering therapeutic agents. For such applications, sEVs can be engineered to display specific moieties on their surfaces (e.g., cell‐targeting antibodies) and to carry therapeutic cargo (e.g., small‐molecule drugs) [19]. These modifications can be achieved through genetic manipulation of the parent cells or through chemical alterations applied post‐isolation [19]. Additionally, sEVs can be fused with synthetic liposomes to create hybrid EVs with enhanced functional properties [20]. Some sEVs also exhibit inherent capabilities particularly relevant to neurodegenerative disease treatment, including organotropism toward the brain [21] and the ability to cross the blood‐brain barrier (BBB) [22], which are two major hurdles for conventional nanoparticle‐based delivery systems.

In this narrative review, we provide a comprehensive and critical synthesis of current evidence on neural cell–derived sEVs and their roles in NDDs, with a particular focus on AD and PD. We examine emerging insights into the involvement of sEVs in disease pathophysiology, highlight recent advances in their use as diagnostic and prognostic biomarkers, and evaluate their growing potential as therapeutic tools and drug delivery systems. In addition, we critically discuss bioengineering strategies designed to enhance sEV stability, targeting specificity, and therapeutic efficacy, with attention to translational relevance.

### 1.1 Literature Search Strategy and Study Selection

Relevant literature was identified through targeted and iterative searches of PubMed, Web of Science, and Scopus, covering publications from database inception through January 2026, with the final literature update performed in January 2026. Searches employed combinations of keywords related to extracellular vesicles and neurodegeneration, including small extracellular vesicles, exosomes, neurons, astrocytes, microglia, oligodendrocytes, neural stem cells, brain endothelial cells, Alzheimer's disease, Parkinson's disease, and drug delivery. Only English‐language articles were considered. Both peer‐reviewed publications and preprints were screened to capture emerging developments, although the final manuscript predominantly reflects peer‐reviewed studies.

Study selection prioritized relevance to neural cell–derived sEVs in NDDs, provision of mechanistic or functional insight, and overall experimental rigor. Beyond general alignment with the MISEV recommendations, additional quality considerations included appropriate sEV characterization, use of biologically relevant in vitro or in vivo disease models, and clarity of experimental design and outcome reporting. The final selection of studies was determined through iterative discussion among the authors, with any disagreements regarding inclusion or interpretation resolved by consensus.

### 1.2 Criteria for sEV Characterization and MISEV Alignment

For the purposes of this review, studies were considered “MISEV‐aligned” if they provided foundational evidence supporting the vesicular nature of the isolated material and its classification as sEVs [7]. At a minimum, this generally included assessment of particle size and size distribution, demonstration of EV‐associated markers, and consideration of potential non‐vesicular contaminants, with the specific methods varying according to study objectives and experimental context. In functional or therapeutic studies, additional emphasis was placed on appropriate experimental controls, biologically relevant models, and clear attribution of observed biological effects to sEV‐associated cargo rather than co‐isolated components. It is acknowledged that the depth of characterization varied across studies, reflecting both the evolution of field‐wide standards and differences in experimental focus.

### 1.3 Data Synthesis, Statistical Reporting, and Analytical Scope

Given the substantial heterogeneity across the literature, including differences in sEV sources, isolation and characterization strategies, disease models, and outcome measures, a formal systematic review, meta‐analysis, or quantitative data synthesis was neither feasible nor appropriate. Instead, findings were integrated using a qualitative, thematic approach, with studies compared based on shared biological mechanisms, cellular origin of sEVs, disease context, and reported functional outcomes, while explicitly considering methodological differences to avoid overgeneralization.

Throughout this review, statistical interpretations are reported as presented in the original studies. Terms such as “significant” are used only where explicitly stated by the original authors. Effect sizes and confidence intervals were not consistently reported across the reviewed literature and are therefore discussed only when available. Where such metrics were not provided, descriptive language has been used to avoid implying unsupported statistical precision.

### 1.4 Considerations Regarding Bias and Limitations of Literature

Several sources of bias should be considered when interpreting the findings summarized in this review. Publication bias may favor studies reporting positive or therapeutic effects of sEVs, particularly in preclinical models, while neutral or negative findings may be underrepresented. In addition, much of the current evidence is derived from in vitro systems and animal models, which may not fully capture the complexity, heterogeneity, and chronic progression of human neurodegenerative disorders.

Methodological heterogeneity represents a further limitation. Variations in sEV isolation techniques, characterization depth, and cargo analysis can influence reported biological effects and complicate cross‐study comparisons. Although increasing adherence to MISEV recommendations has improved standardization, differences in experimental rigor and reporting persist. Together, these considerations highlight the need for cautious interpretation of preclinical findings and underscores the importance of standardized methodologies, transparent reporting, and rigorous validation to support the responsible translation of sEV‐based strategies for neurodegenerative diseases.

## 2 Roles of sEVs in Neurodegenerative Diseases

sEVs are increasingly recognized as important mediators in the pathology of neurodegenerative diseases due to their involvement in key processes such as the intercellular transmission of pathological proteins (e.g., tau and α‐synuclein), propagation of neuroinflammation, modulation of oxidative stress responses, synaptic function, and cellular waste clearance. Given these dual roles, sEVs have attracted significant attention as both potential biomarkers and therapeutic targets in neurodegeneration. In the following sections, we explore their specific contributions in the context of AD and PD.

### 2.1 The Role of sEVs in Alzheimer Disease

AD is a progressive neurodegenerative disorder marked by cognitive decline, particularly memory loss [23]. It is characterized by the accumulation of amyloid‐β (Aβ) plaques outside neurons and tau neurofibrillary tangles (NFTs) within neurons, both of which disrupt neuronal function, ultimately leading to cell death [24].

**FIGURE 2:**
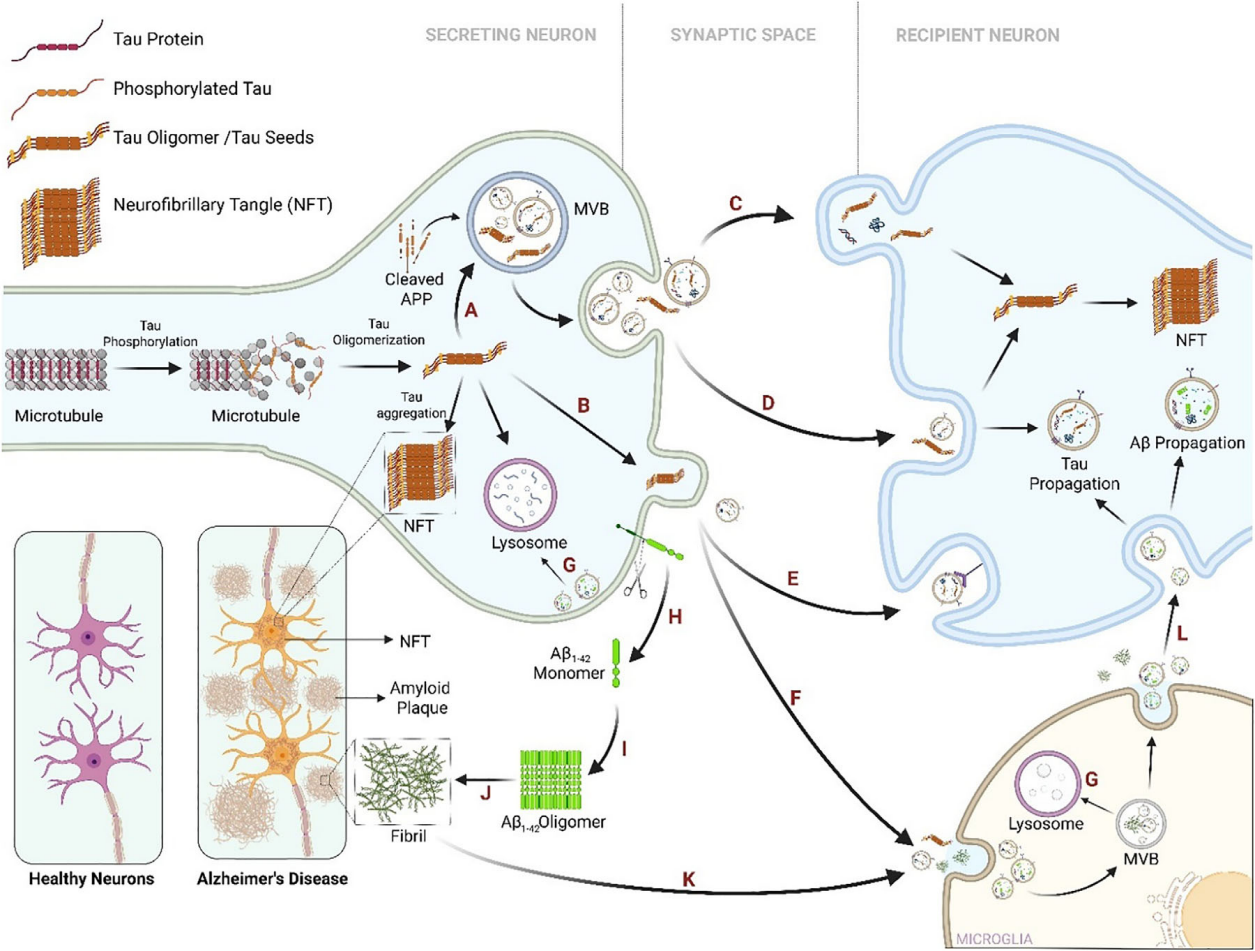
Formation of amyloid plaques and neurofibrillary tangles (NFTs) and the role of sEVs in Alzheimer's disease propagation involving tau proteins and Aβ peptides. Tau is propagated via sEVs either through: (A) Presynaptic encapsulation in multivesicular bodies (MVBs), followed by fusion with the plasma membrane to release tau oligomer–carrying sEVs; (B) or direct plasma membrane budding (ectosomes). Once in the synaptic cleft, tau‐containing sEVs are taken up by recipient neurons via (C) sEVs fusion with plasma membrane; (D) bulk‐endocytosis or (E) macropinocytosis mediated by heparan sulfate proteoglycans. Extracellular tau and tau carrying EVs are also taken up by microglia (F). Under physiological conditions, tau and Aβ (G) encapsulated in sEVs are degraded by lysosomes. Aβ propagation occurs as follows: (H) Amyloid precursor protein (APP) is cleaved to Aβ_1‐42_ by β‐secretase and subsequently by γ‐secretase within the membrane; (I) high Aβ concentrations drive oligomer formation, which aggregate into (J) fibrils, which are the building blocks of amyloid plaques. Microglia attempt fibril clearance by (K) engulfing and encapsulating them in sEVs, which are initially degraded, but later released following MVB fusion with the plasma membrane. (L) Released sEVs and fibrils are either taken up by neurons or accumulate extracellularly, leading to further Aβ propagation. This image was created using Biorender.com.

Tau, a microtubule‐associated protein enriched in neurons, stabilizes microtubules under normal conditions [25]. In AD, tau becomes hyperphosphorylated, dissociates from microtubules, and aggregates into insoluble tangles [26]. These pathological tau species are released extracellularly, where they may activate microglia and propagate via prion‐like mechanisms, as shown in Figure 2A–G. Aβ is a 38–43 amino acid peptide produced by the sequential cleavage of amyloid precursor protein (APP) by β‐ and γ‐secretases. While Aβ may have physiological roles in neurogenesis and neuroprotection [27], the aggregation of its isoforms, particularly the oligomer‐prone Aβ_1–42,_ into oligomers, protofibrils, and fibrils leads to the formation of amyloid plaques, a hallmark of AD [28]. Oligomeric Aβ species are especially neurotoxic due to their ability to disrupt membrane integrity and cellular homeostasis. Microglia initially aid in Aβ clearance; however, chronic activation promotes inflammation, neuronal injury, and accelerates disease progression, as illustrated in Figure 2G–L [29].

While both Aβ and tau contribute to AD pathology, evidence suggests that tau burden correlates more strongly with disease progression [30]. For detailed mechanisms underlying AD pathogenesis, the reader is referred to other comprehensive reviews [31, 32]. In the context of this review, we now examine how sEVs influence AD pathophysiology, particularly in relation to the spread of Aβ and tau.

sEVs have been implicated in both the pathogenesis of AD [33], and as potential biomarkers capable of predicting the progression from Mild Cognitive Impairment (MCI) to overt AD [34]. In line with this, neural cell‐derived sEVs isolated from blood have been shown to carry disease‐relevant biomolecules, enabling the detection of AD with promising accuracy [6, 15, 33, 35, 36]. The accumulation of misfolded proteins such as Aβ and tau is neurotoxic, and the sEV‐mediated transmission of these pathological proteins between neurons has been proposed as a key mechanism underlying the spread of AD pathology within the brain [33, 37].

In the early stages of disease, sEVs may serve a protective role by facilitating the removal of toxic aggregates from affected cells. However, as the disease progresses, they appear to contribute to the dissemination of pathology to neighboring healthy cells [38]. Several studies have shown that sEVs carrying toxic tau and Aβ oligomers are readily internalized by recipient neurons, thereby promoting the spread of neurodegenerative pathology. sEVs containing tau oligomers [33] and Aβ oligomers [37], isolated from AD patient brains, have been shown to be taken up by neuronal cultures. Sardar Sinha et al. demonstrated that Aβ oligomer–laden sEVs were either internalized by or transferred cargo to healthy neurons, resulting in toxicity. Furthermore, inhibition of sEV production, release or uptake significantly reduced the neuron‐to‐neuron transmission of Aβ oligomers (*p* < 0.001) and associated cytotoxicity [37]. Asai et al. further established a link between microglia‐derived sEVs and tau propagation. Their study revealed that tau spread was mediated via microglial sEVs, and that depletion of microglia significantly curtailed sEV production and tau dissemination both in vitro and in vivo (*p* < 0.05–0.0001) [18].

Furthermore, Aβ‐containing sEVs isolated from PSN1 mutant neurons and CSF of sporadic, late‐onset AD patients induce toxicity in primary rat cortical neurons, with a strong correlation between sEV Aβ_1–42_ levels and the induced neurotoxicity [39, 40]. Nogueras‐Ortiz et al. reported that circulating sEVs from AD patients, particularly those derived from astrocytes, triggered complement‐mediated neurotoxicity by delivering complement proteins that facilitated membrane attack complex formation and necroptosis in primary cortical neurons [41]. Joshi et al. found that microglial MVs were significantly elevated in AD patients (*p* < 0.05) and exerted marked neurotoxic effects in neuronal cultures. This effect was linked to MV lipids facilitating the conversion of insoluble Aβ aggregates into soluble, neurotoxic forms, as well as the vesicular transport of toxic Aβ species. Notably, the neurotoxic impact was alleviated when neurons were treated with the Aβ‐binding protein PrP or anti‐Aβ antibodies, both of which prevented harmful Aβ interactions with neuronal membranes [42].

Despite strong evidence supporting the involvement of sEVs in AD, their overall role appears to be dual‐faceted, exhibiting both pathogenic and protective functions. It is imperative to note that only a minor fraction of extracellular Aβ (less than 1%) and Tau (ranging from less than 0.2% to approximately 2%–3%) is associated with sEVs [39]. Nevertheless, while total Aβ_1–40/42_ levels in sEVs isolated from plasma and CSF of AD patients are lower than in EV‐free fractions, the Aβ_1–42_/Aβ_1–40_ ratio is higher in sEVs [39]. This suggests that sEVs, although limited in abundance, may selectively concentrate pathogenic species and thereby contribute meaningfully to AD pathology. Overall, the relationship between sEVs and AD is complex and context‐dependent, warranting further investigation to delineate their dualistic roles in disease propagation and potential neuroprotection [15, 39, 43].

### 2.2 The Role of sEVs in Parkinson Disease

**FIGURE 3:**
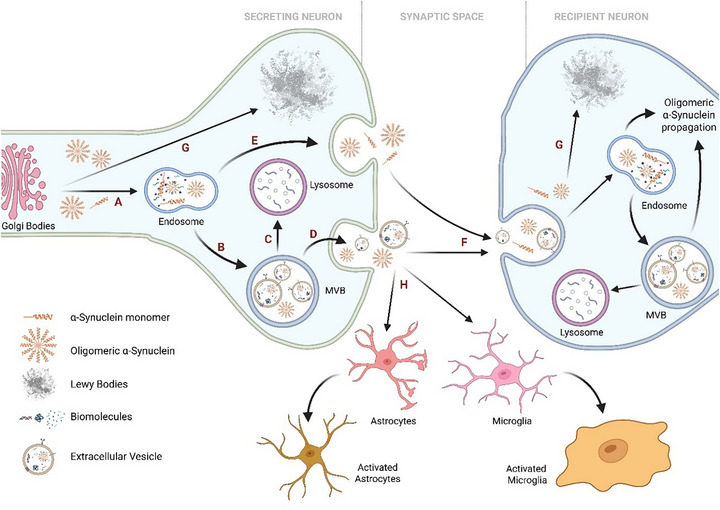
Formation of Lewy bodies and the role of sEVs in α‐synuclein (αS) propagation. In secreting neurons, (A) monomeric and oligomeric αS are sorted into endosomes, which (B) mature into multivesicular bodies (MVBs). Cytoplasmic αS can also enter MVBs via inward budding. MVBs then either (C) fuse with lysosomes for degradation or (D) with the plasma membrane to release αS‐containing sEVs into the synaptic space. (E) Additionally, αS species may be incorporated into secretory vesicles and released via exocytosis. (F) In the synaptic cleft, free and sEV‐associated αS are taken up by recipient neurons through micropinocytosis, promoting further oligomeric αS propagation. (G) Accumulation of oligomeric αS ultimately results in the formation of Lewy bodies and Lewy neurites. (H) Both free and sEV‐encapsulated αS are also internalized by microglia and astrocytes, which initially aid clearance but, with continuous uptake, contribute to astroglial inflammation. This image was created using Biorender.com.

PD is a chronic, progressive neurodegenerative disorder clinically presenting with bradykinesia, resting tremor, rigidity, and postural instability [44]. Pathologically, it is defined by the intracytoplasmic accumulation of misfolded α‐synuclein (αS), which aggregates into Lewy bodies (LBs) and Lewy neurites (LNs) within surviving neurons [45]. EVs contribute to αS propagation by packaging monomeric and oligomeric αS into MVBs and releasing them as sEVs or via exocytosis. In the synaptic cleft, free and sEV‐associated αS are internalized by neurons, promoting oligomer spread. Lysosomal dysfunction and reduced GBA‐1 activity further increase vesicular release, and the ganglioside‐rich environment of sEVs promotes αS aggregation, ultimately leading to LB and LN formation, as shown in Figure 3A–G [46]. Glial cells also internalize αS and αS‐containing EVs; although this initially supports clearance, persistent uptake promotes astroglial and microglial activation, fueling neuroinflammation (Figure 3H) [47].

In addition to vesicle‐mediated αS propagation, several interconnected mechanisms drive PD progression in both familial and sporadic forms. Elevated ROS levels impair mitochondrial function and amplify oxidative damage, while persistent neuroinflammation mediated by activated microglia and astrocytes exacerbates neuronal stress. Excitotoxicity caused by excessive glutamate release further disrupts synaptic homeostasis, and the loss of critical neurotrophic factors reduces neuronal resilience and repair capacity [48]. For broader insights into PD pathogenesis, readers are referred to other comprehensive reviews [49, 50]. Here, we specifically focus on the role of sEVs in the propagation and modulation of PD‐related pathology.

Because αS lacks an endoplasmic reticulum targeting sequence, it was initially considered an exclusively intracellular protein [51, 52]. This view was later challenged by studies detecting extracellular αS in human CSF [51] and plasma [52]. sEVs have since been implicated in the propagation of αS, contributing to PD pathogenesis. Both monomeric and oligomeric forms of αS have been detected on the surface and within the lumen of sEVs. However, similar to tau and Aβ, only a small fraction of total αS is associated with sEVs [53, 54, 55]. Nevertheless, in vitro studies demonstrate that sEVs not only facilitate transcellular transmission of αS, but also reduce neuronal viability in a concentration‐dependent manner [53]. Although oligomeric αS is secreted both freely and via sEVs, the sEV‐associated form is more readily internalized by recipient cells and exhibits greater toxicity than free oligomers [55].

Further evidence links impaired lysosomal function to increased sEV‐mediated αS release. Specifically, blocking intracellular trafficking through lysosomes or inhibiting glucocerebrosidase 1 activity, elevates the release of αS‐laden sEVs, suggesting that lysosomal dysfunction may accelerate PD pathology [56, 57]. Additionally, αS encapsulated within sEVs shows a higher propensity to aggregate, likely due to the abundance of gangliosides in the vesicle membrane, supporting a prion‐like mechanism of spread [58]. Comparative studies of sEVs from PD patients and healthy controls revealed that only PD‐derived sEVs induced αS aggregation, further supporting their pathogenic role [59]. Moreover, inhibition of nSMase‐2 in SH‐SY5Y cells reduced both sEV secretion and the intercellular transfer of oligomeric αS (*p* < 0.01), reinforcing the role of sEVs in disease propagation [60].

Beyond αS transmission, sEVs have also been implicated in the dissemination of PD‐associated pathogenic proteins whose trafficking and release are influenced by genetic risk factors. Mutant forms of leucine‐rich repeat kinase 2 (LRRK2), a gene strongly linked to late‐onset PD, can be internalized into endosomal compartments, incorporated into MVBs, and released via sEVs in a Rab GTPase–dependent manner [61]. Notably, the R1441C LRRK2 mutation induces the formation of enlarged and dysfunctional MVBs, enhancing sEV secretion and promoting the increased release of toxic αS species, thereby accelerating intercellular disease propagation [62]. In addition, genetic variation at the MAPT locus, which encodes the tau protein and represents an established risk factor for PD, may further modulate sEV‐mediated pathogenic signaling. Although tau is not a primary pathological hallmark of PD, it is known to be packaged and transmitted via sEVs in other neurodegenerative contexts, and altered tau expression has been shown to influence αS aggregation and neuronal vulnerability, suggesting that MAPT‐associated risk may indirectly shape sEV cargo composition and downstream neurodegenerative processes [63, 64].

Collectively, evidence from both AD and PD indicates that sEVs exert context‐dependent roles, functioning either as mediators of neurodegenerative pathology or as contributors to neuroprotection. On the pathogenic side, sEVs facilitate the intercellular dissemination of misfolded proteins, including Aβ, tau, and α‐synuclein, thereby promoting prion‐like spread of toxic aggregates across neural networks [6]. Microglia‐derived sEVs, in particular, have been implicated in driving tau and α‐synuclein transmission, while exosome‐enriched preparations from PD patients have been shown to induce protein aggregation, dopaminergic neurodegeneration, microglial activation, and motor deficits in vivo [65]. Conversely, sEVs also participate in protective processes under physiological or early disease conditions, including waste clearance and the delivery of anti‐inflammatory or neurotrophic cargo that counteracts neuronal stress and degeneration [66]. This apparent duality has led to the characterization of sEVs as both “friends and foes” in neurodegeneration. Importantly, discrepancies across studies likely arise from experimental heterogeneity, including differences in sEV cellular origin, cargo composition, disease models, and analytical endpoints, underscoring the need for standardized approaches to more precisely delineate their roles in NDD pathogenesis and therapy.

**FIGURE 4:**
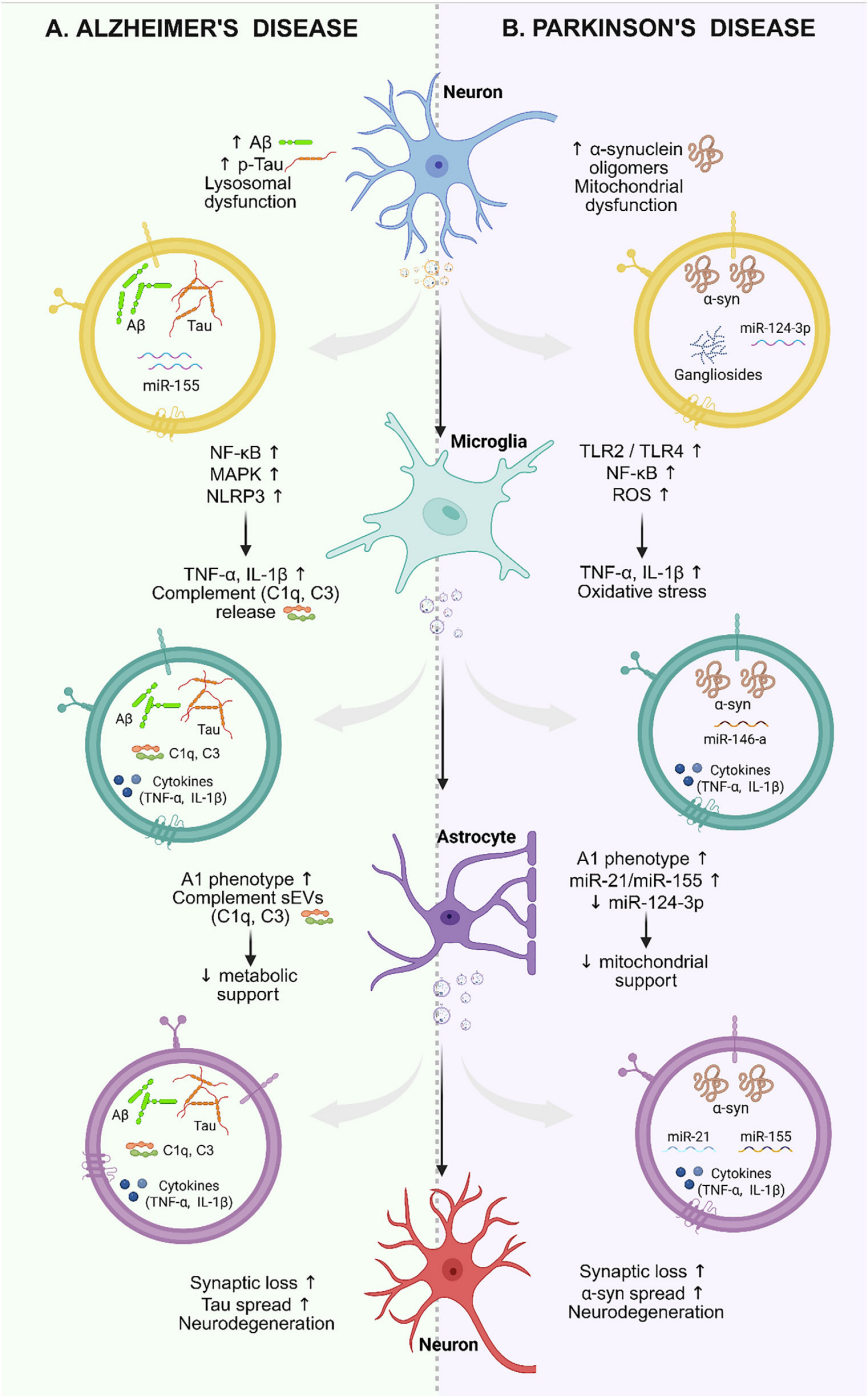
Disease‐specific roles of sEVs in modulating neuroinflammation in (A) Alzheimer's Disease and (B) Parkinson's Disease. (A) In Alzheimer's disease (left panel), neuronal lysosomal dysfunction promotes the accumulation of amyloid‐β (Aβ) and phosphorylated tau and facilitates their incorporation into sEVs. Neuron‐derived sEVs containing Aβ, tau, and pro‐inflammatory microRNAs are internalized by microglia, resulting in activation of NF‐κB, MAPK, and NLRP3 inflammasome signaling pathways, leading to increased cytokine and complement (C1q, C3) release. Microglial‐derived sEVs subsequently propagate Aβ and tau pathology and induce astrocytic reactivity. Reactive astrocytes adopt an A1‐like phenotype and release complement‐enriched sEVs while exhibiting reduced metabolic support, ultimately driving synaptic loss, tau spread, and neuronal degeneration. (B) In Parkinson's disease (right panel), neuronal stress associated with mitochondrial dysfunction leads to increased accumulation of α‐synuclein oligomers and promotes the release of α‐synuclein–containing sEVs enriched with lipid components (e.g., gangliosides) and regulatory microRNAs. Following release, neuronal sEVs are taken up by microglia, where they activate Toll‐like receptor (TLR2/4) signaling, leading to NF‐κB activation, increased reactive oxygen species (ROS) production, and elevated pro‐inflammatory cytokine release (TNF‐α, IL‐1β). Activated microglia subsequently release secondary sEVs carrying inflammatory mediators and α‐synuclein, which are internalized by astrocytes. This uptake drives astrocytic polarization toward a neurotoxic A1‐like phenotype, characterized by increased expression of pro‐inflammatory microRNAs (e.g., miR‐21 and miR‐155), reduced miR‐124‐3p levels, impaired mitochondrial and metabolic support, and enhanced inflammatory signaling. Reactive astrocytes then release pathogenic sEVs that exacerbate synaptic dysfunction, facilitate α‐synuclein spread, and contribute to progressive neurodegeneration. Selected miRNAs are shown as representative examples of regulatory or pro‐inflammatory sEV cargo and are not intended to indicate disease‐ or cell‐type‐specific biomarkers. Their biological effects are context‐, dose‐, and stage‐dependent and may vary according to cellular origin and inflammatory state. This figure was created using Biorender.com.

In this context, Figure 4 provides an integrated mechanistic framework illustrating how neural cell–derived sEVs shape disease‐specific neuroinflammatory cascades in AD and PD. In AD (Figure 4A), neuronal lysosomal dysfunction facilitates the incorporation of amyloid‐β and tau into sEVs, promoting their dissemination and uptake by microglia. This process is accompanied by activation of microglial NF‐κB, MAPK, and inflammasome signaling pathways, leading to pro‐inflammatory cytokine and complement release. Astrocyte‐derived sEVs in AD further amplify neurodegeneration through complement‐mediated synaptic toxicity and reduced neuronal support, thereby contributing to synaptic loss and progressive neuronal dysfunction [67]. In PD (Figure 4B), neuronal sEVs enriched in α‐synuclein and lipid components, together with regulatory inflammatory cargo, promote microglial activation through TLR‐dependent signaling, resulting in oxidative stress and pro‐inflammatory cytokine production. Subsequent microglial sEV signaling reprograms astrocytes toward a reactive A1‐like phenotype, impairing metabolic support and exacerbating synaptic vulnerability and α‐synuclein propagation [68]. Together, these mechanisms underscore how sEVs act as context‐dependent regulators of neuroinflammation, with their pathological impact shaped by cellular origin, cargo composition, disease stage, and inflammatory context.

It is important to note here that studies investigating the role of sEVs in AD and PD employ diverse experimental models, including transgenic animals, toxin‐induced models, and in vitro systems, which contribute to variability in reported outcomes. Differences in disease stage, cellular source of sEVs, and experimental context underlie much of this discordance. Accordingly, findings are interpreted in this review by emphasizing shared mechanistic trends, such as sEV‐mediated propagation of pathogenic proteins, modulation of neuroinflammation, and context‐dependent neurotoxic or neuroprotective effects, while explicitly acknowledging model‐dependent differences and avoiding direct outcome comparisons across heterogeneous systems. Against this pathophysiological backdrop, growing evidence now supports the therapeutic exploitation of neural cell–derived sEVs, either as biologically active agents or as platforms for targeted intervention in neurodegenerative diseases.

## 3 Neural sEVs as a Therapeutic Tool in the Treatment of NDDs

### 3.1 Source, Context, and Model Dependent Effects of Neural sEVs

The cellular origin of sEVs is a major determinant of their functional impact, as vesicles released from different neural cell types or from the same cell type under distinct activation states can exert markedly different effects. Under physiological conditions, astrocyte‐derived sEVs often carry neuroprotective cargo, such as apolipoprotein D, supporting neuronal survival and homeostasis [69]. In contrast, sEVs released from reactive astrocytes or activated microglia may be enriched in inflammatory cytokines or toxic proteins that exacerbate neuronal injury [70]. Neuron‐derived sEVs can also contribute to disease propagation by transporting pathological tau or α‐synuclein species that seed aggregation in recipient cells [71]. Although glia‐derived sEVs frequently play supportive roles by delivering metabolic enzymes, growth factors, or antioxidant proteins, this homeostatic function can shift toward deleterious signaling when parent cells adopt a chronically reactive phenotype [72].

Divergent findings across studies are further shaped by the experimental models employed. In vitro systems and toxin‐based PD models, such as MPP^+^ or 6‐OHDA exposure, primarily capture acute neuronal stress and cell death, and often reveal protective effects of glial sEVs that attenuate apoptosis [73]. By contrast, transgenic models of AD and PD, which recapitulate chronic protein aggregation and sustained neuroinflammation, highlight roles for sEVs in long‐term processes, including synaptic dysfunction, gliosis, and behavioral impairment [74]. Variability also arises from methodological differences, including sEV isolation techniques, dosing regimens, and routes of administration [75]. While some studies administer purified sEVs intravenously or intranasally as therapeutic agents, others focus on endogenous brain‐derived or circulating sEVs as mediators of pathology, complicating direct comparisons.

Despite this heterogeneity, several mechanistic themes have emerged. Neuroprotective actions of neural sEVs are frequently attributed to their microRNA and protein cargo, which modulate cell survival and inflammatory signaling pathways. For example, astrocyte‐derived sEVs containing miR‐200a‐3p suppress pro‐apoptotic MKK4 signaling in neurons (*p* < 0.01), thereby protecting against MPP^+^‐ and glutamate‐induced toxicity via inhibition of the JNK pathway [76]. Similarly, microglia‐derived sEVs enriched in miR‐124‐3p or miR‐711 can promote anti‐inflammatory microglial polarization, reduce tau hyperphosphorylation, and improve cognitive outcomes in rmTBI AD mouse models (*p* < 0.05–0.001) [77, 78].

Conversely, pathogenic effects of sEVs are most often linked to their capacity to disseminate misfolded proteins and inflammatory signals. α‐synuclein loaded sEVs released from neurons or microglia can be readily internalized by recipient cells, where they nucleate new aggregates and amplify pathology [58]. In parallel, sEV‐associated cytokines and inflammasome components can activate neighboring glial cells, reinforcing feed‐forward inflammatory loops [79]. Importantly, the same neural cell type may generate either protective or harmful sEVs, depending on its activation state and microenvironment. Resting microglia, for instance, may release vesicles that facilitate debris clearance and Aβ removal, whereas chronically activated microglia secrete sEVs enriched in proteases and inflammatory mediators that accelerate neuronal damage [72].

Taken together, the literature portrays neural cell–derived sEVs as a double‐edged component of AD and PD pathophysiology. They can propagate neurotoxic protein aggregates and inflammatory signaling, thereby contributing to disease progression, yet they also deliver protective cargo that supports neuronal survival, modulates immune responses, and facilitate repair. Many apparent inconsistencies across studies can be attributed to variability in sEV source, cargo composition, experimental design, and outcome measures. Addressing these variables through standardized methodologies and systematic comparisons will be essential for clarifying how sEV origin and context dictate function. Ultimately, the challenge and opportunity lies in selectively harnessing the beneficial properties of sEVs, while limiting their pathogenic potential.

### 3.2 Neural Cell‐Derived sEVs in the Treatment of NDDs

Isolation of sEVs has been successfully demonstrated across different neural cell types, encompassing NSCs [80, 81, 82], neurons [83, 84] astrocytes [85] microglia [86] and oligodendrocytes [87]. Despite this broad technical feasibility, the translation of neural cell–derived sEVs into effective therapeutic agents or drug delivery systems (DDS) for NDDs remains comparatively limited. To date, the predominant focus of research has been on elucidating the roles of sEVs in disease progression [70, 88, 89, 90] and their utility as diagnostic or prognostic biomarkers [91, 92, 93], with relatively fewer studies rigorously evaluating therapeutic efficacy under conditions that reflect disease chronicity, aging, and clinical heterogeneity.

Beyond their role as intercellular transporters, neural cell–derived sEVs exert context‐dependent biological effects through their molecular cargo. Under physiological conditions, sEV‐associated proteins and microRNAs contribute to neural cell–to–cell communication, regulation of synaptic function, and maintenance of cellular homeostasis [94, 95]. In pathological settings, however, disease‐associated alterations in sEV cargo composition can drive neurodegenerative processes. Proteins such as tau, amyloid‐β, and α‐synuclein packaged within sEVs have been implicated in the intercellular propagation of pathogenic protein aggregates in Alzheimer's and Parkinson's disease [96, 97]. In parallel, miRNAs carried by neural sEVs regulate gene expression in recipient cells and can modulate pathways related to neuroinflammation, synaptic plasticity, mitochondrial homeostasis, and protein aggregation [98]. Importantly, these same cargo‐dependent mechanisms can be therapeutically leveraged, as sEVs enriched either naturally or through engineering with neuroprotective proteins or regulatory miRNAs have demonstrated the capacity to suppress pathological signaling pathways and improve functional outcomes in preclinical models [38]. Collectively, these findings underscore that variations in sEV cargo composition, influenced by cellular origin and disease state, are key determinants of whether sEVs promote disease progression or confer therapeutic benefit.

Consistent with this concept, studies indicate that sEVs derived from healthy neural cells not only support intercellular communication but can also exert protective effects on compromised neural cells [69, 77, 81]. However, much of this evidence is derived from acute injury or toxin‐based models, which may not fully recapitulate the progressive, multifactorial nature of human NDDs. Among the various neural sources, sEVs isolated from NSCs have received the greatest attention and are the most extensively studied in the context of NDD intervention. Accordingly, the following sections examine neural cell–derived sEVs as therapeutic tools, beginning with NSC‐derived sEVs, which currently represent the most well‐characterized and extensively investigated source.

#### 3.2.1 Neural Stem Cell‐Derived sEVs

NSCs are multipotent progenitor cells present in both developing and adult brains, capable of self‐renewal and differentiation into neurons, astrocytes, and oligodendrocytes. They originate from fetal, postnatal, and adult brain tissue, as well as from embryonic stem cells and induced pluripotent stem cells. Primary NSCs, a subtype of radial glial cells, give rise to intermediate progenitors via asymmetric division [99]. Their therapeutic potential has been demonstrated in studies involving transplantation into aged or injured brains [100]. While NSC‐based cell therapies show promise, increasing attention has shifted toward cell‐free approaches using NSC‐derived products, particularly sEVs, in part to mitigate risks associated with graft survival, tumorigenicity, and immune rejection.

NSC‐derived sEVs exhibit distinct cargo composition and targeting behavior compared to sEVs from other stem or somatic cells. These vesicles are enriched with miRNAs and proteins implicated in neural regeneration, neuroprotection, and plasticity [101]. Moreover, hypothalamic NSC‐derived sEVs appear to influence aging‐related processes, potentially acting as independent metabolic units [102, 103]. When administered exogenously, either intravenously or intranasally, NSC‐derived sEVs can modulate the brain microenvironment and enhance neural function under both physiological and pathological conditions. Nevertheless, the biodistribution, cellular uptake specificity, and long‐term persistence of these effects remain incompletely characterized across disease stages.

Multiple investigations have assessed the therapeutic potential of NSC‐derived sEVs in AD models. In a transgenic AD mouse study, Apodaca et al. demonstrated that intravenous administration of human NSC‐derived sEVs in a 5xFAD transgenic AD mouse model improved cognitive outcomes, restored fear extinction memory, reduced anxiety‐like behavior (*p <* 0.01), and attenuated neuropathology through decreased Aβ plaque load and microglial activation (*p <* 0.05) [104]. While these findings are compelling, the reliance on a single transgenic mouse model and short‐term behavioral endpoints limits conclusions regarding longevity of the therapeutic response and disease‐modifying capacity. Similarly, Li et al. investigated the effects of sEVs derived from non‐transgenic embryonic mouse NSCs in an APP/PS1 transgenic mouse model of AD. Treatment with these sEVs enhanced mitochondrial function and activated the SIRT1 pathway, which is closely linked to cellular energy metabolism and neuroprotection. The intervention also promoted synaptic activity, suppressed neuroinflammatory responses, and ultimately improved cognitive performance (*p <* 0.01) in treated mice, highlighting the multifaceted therapeutic potential of NSC‐derived sEVs in AD [105]. Gao et al. investigated sEVs from induced NSCs (iNSCs) and compared them to those isolated from primary NSCs. IV administered sEVs crossed the BBB and were internalized by neurons, astrocytes, and microglia. Both NSC‐EVs and iNSC‐EVs significantly improved spatial learning and memory, reduced Aβ plaque burden and phosphorylated tau levels, enhanced dendritic spine density, and restored neurogenesis in 5xFAD AD mouse model (*p <* 0.05–0.001). sEV treatment also reduced microgliosis and astrogliosis, suppressing pro‐inflammatory proteins (CD86, iNOS, IL‐1β). Mechanistically, transcriptomic and miRNA profiling revealed enrichment of let‐7, miR‐9, miR‐21, and miR‐10b families in sEVs, which likely mediated downregulation of pro‐inflammatory and Aβ production‐related genes. Importantly, iNSC‐EVs displayed therapeutic efficacy equal or superior to NSC‐EVs and outperformed MSC‐ or fibroblast‐derived sEVs, highlighting their translational promise as a scalable and ethically favorable alternative for AD therapy [106]. Despite these strengths however, therapeutic superiority was assessed within a single disease context, and scalability claims remain inferential without GMP‐aligned production data.

Attaluri et al. further demonstrated that intranasally administered hiPSC‐derived NSC‐sEVs (hiPSC‐NSC‐sEVs) rapidly distributed throughout the brain, predominantly targeting neurons and microglia in 5xFAD mice (*p <* 0.05–0.001). Treatment suppressed neuroinflammatory cascades and reduced both Aβ plaque load and phosphorylated tau levels. This demonstrated that hiPSC‐NSC sEVs can ameliorate AD‐like neuroinflammation and proteopathic lesions in vivo [107]. In a related study, Madhu et al. further showed that intranasally delivered hiPSC‐NSC‐sEVs in 5xFAD mouse model modulated glial responses and reprogrammed disease‐associated microglia and astrocytes. These effects included downregulation of NLRP3 inflammasome components, IFN‐1 signaling, and IL‐6 expression (*p <* 0.05–0.01). Functional improvements in cognition and mood were observed and persisted for up to two months, alongside reductions in plaque burden, astrocyte hypertrophy, and p‐tau accumulation [108]. While intranasal delivery offers translational appeal, variability in dosing efficiency and nasal‐brain transport across species remains a key translational uncertainty. Additionally, NSC‐derived sEVs have also been shown to restore mitochondrial homeostasis in AD models. In APP/PS1 mice, sEV treatment upregulated SIRT1 expression and activated the SIRT1–PGC1α pathway, promoting mitochondrial biogenesis through increased expression of factors such as NRF1 and COXIV (*p <* 0.01). This intervention enhanced mitochondrial function and reduced astrocyte activation, although it did not affect Aβ production. Spatial mapping further revealed that sEVs corrected AD‐associated disruptions in the distribution of mitochondrial biogenesis markers across brain regions, highlighting their role in enhancing cellular resilience in AD [109]. Notably, these benefits occurred independently of changes in Aβ production, suggesting that NSC‐sEVs may primarily enhance cellular resilience rather than directly modifying upstream amyloidogenic processes.

In PD models, NSC‐derived sEVs have also demonstrated neuroprotective effects. Lee et al. showed that sEVs from NSCs (F3 cells) and fibroblasts prevented 6‐hydroxydopamine (6‐OHDA)–induced neurotoxicity both in vitro and in vivo. sEV treatment reduced oxidative stress, caspase activation, and inflammation (*p <* 0.05–0.0001), and preserved dopaminergic neurons. The sEVs carried regulatory miRNAs, such as miR‐182‐5p, miR‐183‐5p, miR‐9, and let‐7, which likely contributed to the observed antioxidative and anti‐inflammatory effects [110]. Further supporting these findings, Díaz Reyes et al. reported that unmodified NSC‐derived sEVs enriched in catalase conferred robust protection in PD cell models. In MPP^+^ and rotenone‐induced toxicity assays, sEVs reduced oxidative stress and apoptosis, likely through catalase‐mediated ROS scavenging. These results demonstrate that NSC‐sEVs can deliver endogenous antioxidant proteins across the BBB and protect dopaminergic neurons, offering a promising cell‐free therapeutic strategy for oxidative neurodegeneration [111]. Although these toxin‐based models are valuable however, they incompletely capture the α‐synuclein–driven pathology and disease progression observed in human PD.

In summary, NSC‐derived sEVs demonstrate consistent neuroprotective and immunomodulatory effects across AD and PD models. However, the majority of supporting evidence is derived from short‐term studies employing highly controlled preclinical systems, such as single cell types or simplified disease models, highlighting the need for longitudinal, comparative, and mechanistically defined investigations that delineate causal pathways of action before clinical translation can be fully justified.

#### 3.2.2 Astrocytes‐Derived sEVs

Astrocytes, a major type of glial cell, are essential for preserving neural structure and function. Under physiological conditions, astrocyte‐derived sEVs exert neurotrophic and neuroprotective effects, supporting neuronal survival and overall brain homeostasis [70]. Under stress conditions, such as ischemia, oxidative damage, nutrient deprivation, or thermal stress, astrocytes release sEVs enriched with survival‐promoting factors. These sEVs help protect neurons from neurotransmitter‐induced toxicity, support neuronal survival, and promote neurite outgrowth [112, 113, 114, 115], making them promising candidates for NDDs therapy.

Chun et al. investigated the effects of astrocyte‐derived sEVs on human iPSC‐derived cortical neurons and found that these vesicles were efficiently internalized by neurons, leading to significant reductions in apoptosis and cellular senescence. Although they had minimal influence on neurite or axon branching, astrocyte sEVs markedly improved electrophysiological properties, enhancing repetitive action potential firing, its frequency, repolarization speed, and resting membrane potential (*p <* 0.0003–0.0001). Proteomic analysis of the sEVs revealed an enrichment of proteins involved in neuroprotection and neuronal excitability, including HSP90AB1, LRP1, APOE, KCTD12, and G6PD. These findings suggest that astrocyte‐derived sEVs not only enhance neuronal viability, but also promote functional maturation, highlighting their potential utility in therapeutic applications [116]. While functionally informative, however, this in vitro system does not capture neuron–glia network dynamics or immune interactions present in vivo; hence, the results should be extrapolated with caution.

Leggio and colleagues conducted a study wherein they utilized sEVs extracted from astrocytes originating from the ventral midbrain (VMB) and striatum (STR), known sites of degeneration in PD, and compared their neuroprotective effects in PD. sEVs were isolated from astrocytes both under basal conditions and following treatment with the CC chemokine ligand 3 (CCL3), a well‐established neuroprotectant. Using the H_2_O_2_ PD model, the study demonstrated that sEVs derived from both VMB and STR astrocytes ameliorated H_2_O_2_‐induced caspase‐3 activation. Notably, however, sEVs derived from CCL3‐treated astrocytes exhibited a higher neuroprotective effect compared to those from basal astrocytes (*p <* 0.05–0.0001). In the MPP^+^ PD model, it was observed that sEVs derived from nigrostriatal astrocytes countered mitochondrial complex I function impairment. However, only sEVs derived from VMB astrocytes fully restored ATP production in neurons [117]. These findings highlight astrocyte heterogeneity but also indicate that therapeutic efficacy may depend strongly on the anatomical origin and treatment condition of the donor cells. Shakespear et al. further explored the effect of astrocyte‐derived sEVs on MPP+‐induced toxicity, a well‐established in vitro PD model. sEVs significantly reduced neuronal cell death and suppressed MKK4 expression (*p <* 0.01), a key regulator of the apoptotic c‐Jun N‐terminal kinase pathway, in both SH‐SY5Y and mesencephalic cultures. Additionally, these sEVs protected hippocampal neurons from glutamate‐induced excitotoxicity. The protective effects were linked to the presence of miR‐200a‐3p within the sEVs, and direct treatment with this microRNA replicated the neuroprotective effects, confirming its functional significance [76]. Although a mechanistically robust analysis was performed however, the lack of in vivo validation limits assessment of translational relevance.

Collectively, astrocyte‐derived sEVs exhibit neuroprotective capabilities, but variability in astrocyte states, regional origin, and disease context represents a critical challenge for standardizing their therapeutic application.

#### 3.2.3 Microglia‐Derived sEVs

Microglia, the resident macrophages of the brain, are central to CNS development and tissue homeostasis through the regulation of basal inflammatory activity. They act at the intersection of the nervous and immune systems in both physiological and pathological conditions [118]. Microglia‐derived sEVs mediate communication with neural cells and propagate cytokine‐driven inflammatory responses across brain regions [118]. When derived from healthy or anti‐inflammatory microglial phenotypes, these sEVs hold promise as therapeutic agents for neurodegenerative diseases.

Lemaire et al. utilized an in vitro leech CNS model to elucidate the contribution of sEVs derived from microglial cells in the regeneration of injured axons of neurons. They demonstrated the dependency of axon regeneration effectors and mediators on sEVs derived from microglial cells. To identify the molecular drivers of this communication, the team profiled the miRNA cargo of these vesicles and identified six candidates, including miR‐1860, miR‐1705, miR‐2284y‐6, miR‐146a, miR‐858, and miR‐7718. These miRNAs were shown to play pivotal roles in neuronal repair and neurite outgrowth, not only in the leech CNS model, but also in assays using rat primary neurons [86]. While these effects indicate that microglial sEVs promote neuronal regeneration across species, the use of a leech CNS model necessitates caution when extrapolating to mammalian neurodegeneration.

Li et al. investigated the therapeutic effects of sEVs derived from M2‐polarized microglia (M2‐sEVs) in AD. In vitro, M2‐sEVs were efficiently taken up by Aβ_1‐42_–challenged hippocampal neurons, where they improved cell viability, restored mitochondrial membrane potential, reduced intracellular and mitochondrial ROS, and downregulated excessive mitophagy markers (LC3‐II, Beclin1, PINK1, Parkin) (*p <* 0.05–0.001). In vivo, systemic delivery of M2‐SEVs to APP/PS1 mice significantly reduced Aβ plaque burden and oligomer levels, while normalizing aberrant PINK1/Parkin‐mediated mitophagy (*p <* 0.001). These interventions led to improved neuronal integrity and reduced mitochondrial dysfunction. The findings highlight M2‐SEVs as a potential therapeutic avenue in AD, acting through the regulation of mitochondrial quality control and neuroinflammation [119].

On the other hand, Wang et al. compared microglia‐derived large extracellular vesicles (LEVs; >200nm) with sEVs and demonstrated that LEVs exert potent protective effects against AD pathology. In vitro, LEVs significantly inhibited Aβ_40_ and Aβ_42_ fibrillation, suppressed Rac1 activation, stabilized intracellular calcium homeostasis, reduced ROS production, and protected SH‐SY5Y cells from Aβ‐induced cytotoxicity (*p* < 0.01–0.001), effects not observed with sEVs, which instead promoted aggregation. Intranasal administration of LEVs to APP/PS1 mice significantly reduced Aβ plaque burden, improved cognitive function in behavioral tests, and attenuated microglial activation (*p* < 0.05–0.001). Proteomic analysis revealed that LEVs are selectively enriched in neuroprotective proteins, including HSP70, LAMC1, and SIL2B, with SIL2B identified as a key mediator of their anti‐aggregation activity. This study highlights LEVs as a unique EV subtype with strong therapeutic potential for AD [120]. However, these findings are based primarily on in vitro assays and a single transgenic mouse model using EVs derived from immortalized microglial cells, with relatively small sample sizes and preliminary proteomic characterization, which may limit mechanistic interpretation, translational relevance, and scalability of LEV‐based therapies. On the contrary, this divergence also highlights functional heterogeneity among EV subtypes and cautions against assuming uniform therapeutic effects across vesicle classes.

Overall, these studies demonstrate that microglia‐derived vesicles can influence neurodegenerative pathology through modulation of inflammation, axonal repair, and pathogenic protein handling. However, the contrasting efficacy observed between sEVs and LEVs indicates that therapeutic benefit is strongly shaped by vesicle subclass and cargo, rather than microglial origin alone, underscoring the need for subclass‐specific evaluation in translational development.

#### 3.2.4 sEVs Derived from Neurons and other Glial Cells

In addition to neural stem cells, astrocytes, and microglia, other mature neural cells, including neurons, oligodendrocytes, and cerebral endothelial cells, also release sEVs that contribute to central nervous system maintenance and repair. Ghidoni et al. identified cystatin C as a key component of neuron‐derived sEVs, with potential implications for AD pathology. Using primary cortical neurons, the authors demonstrated that cystatin C, together with APP metabolites and presenilin fragments, is secreted via sEVs, rather than solely through the classical secretory pathway. Proteomic analysis revealed nine cystatin C glycoforms enriched in sEVs, while AD‐linked presenilin‐2 mutations (M239I, T122R) significantly reduced both cystatin C and APP/Aβ metabolites (*p <* 0.05–0.001). As cystatin C inhibits amyloid aggregation and supports NSC proliferation, these findings suggest that presenilin mutations disrupt protective sEV‐mediated signaling, promoting neurodegeneration [121]. Peng et al. explored the role of neuron‐derived sEVs in modulating microglial function. Using sEVs isolated from rat cortical neurons, they found that neuron‐derived sEVs, but not LEVs, improved microglial viability up to 0.3‐fold by reducing apoptosis. These sEVs also downregulated microglial activation markers (CD11b, MHC‐II, CD32) and pro‐inflammatory mediators (iNOS, TNF‐α, IL‐6, MCP‐1), while simultaneously increasing the anti‐inflammatory cytokine IL‐10 [122]. Collectively, these results highlight neuron‐derived sEVs as critical modulators of neuron–microglia communication, with potential implications for disease modulation; however, at the current time, these implications remain limited to acute inflammatory paradigms.

Cerebral endothelial cells (CECs) are specialized cells that line the BBB and play a vital role in maintaining brain homeostasis and regulating the transport of substances between the bloodstream and the CNS [123]. sEVs released by BECs represent an emerging area of interest in brain research and may be particularly relevant in the pathophysiology and modulation of NDDs, such as AD and PD. Wang et al. demonstrated that CEC–derived sEVs (CEC‐sEVs) exert strong neuroimmune‐modulatory effects through the delivery of miR‐672‐5p. In LPS‐activated microglia, CEC‐sEVs suppressed pro‐inflammatory cytokines and promoted anti‐inflammatory markers (IL‐10, ARG‐1, CD206). Mechanistically, miR‐672‐5p directly targeted TAB2, disrupting the TAB2–TAK1 interaction and attenuating NF‐κB activation. This also enhanced autophagic flux and promoted degradation of the NLRP3 inflammasome. Systemic administration of CEC‐sEVs in LPS‐challenged mice improved cognition, reduced neuroinflammation, and prevented neuronal apoptosis (*p <* 0.01). Conversely, knockdown of miR‐672‐5p in sEVs abolished these effects, confirming its central role in mediating the therapeutic response [124]. Although promising, however, it is important to note that systemic inflammation models may not fully capture NDD‐specific immune dysregulation.

Oligodendrocytes, the myelin‐forming glial cells, release sEVs carrying bioactive molecules that regulate neuronal activity and immune responses [125]. Under inflammatory conditions, these sEVs can propagate pro‐inflammatory signals and exacerbate demyelination [126], whereas in repair states, they transport factors that support remyelination and myelin sheath regeneration [127]. In a study, Krämer‐Albers et al. showed that cultured oligodendrocytes secrete sEVs rich in structural and stress‐protective proteins, including PLP, 2′,3′‐cyclic‐nucleotide‐phosphodiesterase, myelin basic protein, and myelin oligodendrocyte glycoprotein. Release of these vesicles was upregulated by calcium‐ionophore stimulation, suggesting a mechanism through which oligodendrocyte‐derived sEVs may contribute to axonal support and CNS maintenance [87]. However, their dual capacity to propagate inflammatory signals under pathological conditions necessitates careful context‐specific evaluation. Table 1 summarizes the reported neural sEVs used in the treatment of NDDs.

**TABLE 1:** Summary of the therapeutic modalities using Neural cells derived sEVs.

Cell Type	sEV Source	Active Component	In vitro Model/Design used	In vivo Model/Design used	Target Disease/ Pathways	Therapeutic Outcome	Key Stats Reported?	Limitations	Ref
Neural Stem Cells	human neural stem cell (hNSC)	hNSC‐derived sEVs	NA	5×FAD accelerated transgenic mouse model of AD (early & late stages)	Alzheimer's Disease	↓ Aβ plaque, microglial activation and neuroinflammation, ↑ synaptophysin Improved synaptic function, Improved cognition and anxiety‐like behaviour	Yes	Male mice only; partial cognitive rescue; limited dosing/long‐term assessment; causal sEV cargo not fully defined	[104]
	Hippocampal Cells of non‐transgenic B6C3 mouse embryos	NSC‐derived sEVs	Primary neuronal cultures exposed to Aβ	APP/PS1 Transgenic mouse model of AD	Alzheimer's Disease; neuronal mitochondrial dysfunction	Improved spatial learning and memory; ↑ synaptic proteins (synaptophysin, PSD‐95); Restored mitochondrial function; ↓ neuroinflammation	Yes	Single AD model; invasive delivery route; limited evaluation of sEV biodistribution and long‐term efficacy	[105]
	NSCs, iNSCs (fibroblast‐reprogrammed); MSCs & fibroblasts	NSC‐sEVs iNSC‐sEVs	Uptake assays in neurons, astrocytes, microglia	5×FAD AD mouse model	Alzheimer's disease	Improved cognition, ↓ Aβ, ↓ pTau, ↑ dendritic density, suppressed microgliosis/astrogliosis, miRNAs mediated immunomodulation; iNSC‐EVs ≥ NSC‐EVs, > MSC/FB‐EVs	Yes	Single AD model; key causal miRNA drivers not fully dissected	[106]
	Human iPSC‐derived neural stem cells (hiPSC‐NSCs)	hiPSC‐NSC‐derived sEVs	NA	5×FAD AD mouse model	Alzheimer's disease	Rapid incorporation in neurons and microglia Extensive brain distribution	Yes	Biodistribution study only; therapeutic efficacy not tested	[107]
	hiPSC‐NSCs	hiPSC‐NSC‐derived sEVs	In vitro microglia exposure, Phagocytosis assay	5×FAD AD mouse model	Alzheimer's disease	Downregulation of DAM and Inflammasome genes Suppressed astrocyte hypertrophy, ↓ plaques, ↓ p‐tau preserved cognition & mood; effects lasted ≥2 months	Yes	Limited to one age window; long‐term dosing not evaluated	[108]
	Mouse NSCs	NSE‐derived sEVs	HT22 hippocampal neurons ± Aβ_25–35_ Assays for mitochondrial membrane potential	SIRT1 conditional knockout APP/PS1 mouse (SKO‐AD) model	Alzheimer's disease	↑ mitochondrial membrane potential, ↑ SIRT1–PGC1α pathway activity improved cognition ↓ astrocyte activation modest effect on Aβ burden	Yes	Invasive intracerebroventricular delivery reduces translational relevance	[109]
	Human fibroblasts and human F3 cells	hNSC‐derived sEVs	SH‐SY5Y ± 6‐OHDA; BV2 microglia inflammatory assays	6‐ODHA induced in vivo mouse models	Parkinson's Disease	↑ dopaminergic neuron survival; ↓ ROS and apoptosis; ↓ microgliosis / astrogliosis; ↓ pro‐inflammatory cytokines; miRNA‐mediated neuroprotection	Yes	Acute toxin‐based PD model; invasive intracerebral delivery; limited behavioral rescue and long‐term outcome assessment	[110]
	Mouse NSCs	NSE‐derived sEVs	SH‐SY5Y neuroblastoma PD models 6‐ODHA PD Model Cell viability assays	NA	Parkinson's Disease	Restored viability in α‐syn WT/A53T & 6‐OHDA neurons ↓ apoptosis/necrosis, ↓ ROS catalase in EVs was functional, acting as a natural antioxidant delivery system	Yes	No in vivo PD validation	[111]
Astrocytes	Human astrocytes	Astrocyte‐derived sEVs	Neuronal apoptosis, senescence, neurite outgrowth models	NA	Neurodegene‐ration associated with NDDs	↓apoptosis/senescence, enhanced electrophysiological maturation	Yes	In vitro only Lacks in vivo validation of delivery, efficacy, and safety	[116]
	Mouse primary astrocytes	sEVs isolated from basal and CC chemokine ligand 3 (CCL3) treated astrocytes	H_2_O_2_ PD Model MPP^+^ PD Model	NA	Parkinson's Disease	↓ in H_2_O_2_ induced caspase‐3 activation. Improved neuronal mitochondrial complex I function. Full restoration of ATP production.	Yes	No in vivo validation; toxin‐based cellular model; astrocyte regional specificity limits generalization; cargo drivers not fully resolved	[117]
	Mouse primary astrocytes	Astrocyte‐derived sEVs	MPP+‐induced apoptotic cell death model in SHSY‐5Y cells and primary mesencephalic dopaminergic neurons	NA	Parkinson's disease	Neuroprotective effect through down‐regulation of MKK4 via miR‐200a‐3p targeting ↓ MPP^+^‐induced caspase‐3 activation ↑ neuronal viability in SH‐SY5Y and primary dopaminergic cultures	Yes	In vitro only; toxin‐based models; no in vivo validation; contribution of non‐miRNA cargo not excluded	[76]
Microglia	Leech CNS derived Microglia	sEVs derived from Leech Microglial cell culture	Rat Primary Neuron Model Leech CNS model	N/A	Neural Protection	sEVs induced miRNA dependent axon repair and promoted neurite outgrowth	Yes	In vitro only; cross‐species model (leech EVs → rat neurons); no mammalian microglia EV validation; miRNA targets largely predictive (limited causal validation)	[86]
	Mouse Microglial (BV2) cell line	M2 Microglia‐derived sEVs	HT‐22 hippocampal neurons ± Aβ_1‐42_ Cell Viability, mitochondrial membrane potential and mitophagy marker assays	APP/PS1 AD mouse model	Alzheimer's disease	Restored mitochondrial membrane potential ↓ROS, reversed Aβ‐induced cell death. ↓Aβ plaques and oligomers Suppressed PINK1/Parkin pathway hyperactivation, normalized mitophagy, and alleviated neuronal impairment in APP/PS1 mice.	Yes	Artificial M2 polarization may not reflect in vivo microglia states	[119]
	Human Microglial (HMC3) cell line	LEVs and sEVs derived from microglia	SH‐SY5Y ± Aβ_40/42_ aggregation, ROS signaling assays, apoptosis (Annexin V/PI)	APP/PS1 AD mouse model	Alzheimer's disease	Inhibited Aβ fibrillation, ↓Aβ plaques, Improved cognition, ↓ Rac1 activation, Ca^2^^+^ dyshomeostasis, ROS Macrosomes > small EVs	Yes	Immortalized microglial cell line used; EV yield and scalability not addressed; macrosomes not yet standardized as a therapeutic EV class; limited long‐term safety assessment	[120]
Neurons	Mouse primary cortical neurons	Neuron‐derived sEVs	Mouse cortical neurons expressing PS2 WT or mutants	NA	Alzheimer's disease	↓ APP/Aβ in sEVs	Yes	In vitro only; lacks functional rescue experiments	[121]
	Rat primary cortical neurons	sEVs and LEVs derived from neurons	Primary microglia ± sEVs or LEVs Apoptosis & cytokine assay	NA	Microglia activation & neuroinflammation associated with NDDs	sEVs suppressed pro‐inflammatory cytokines ↑ IL‐10 improved neuronal survival	Yes	Lacks in vivo confirmation of microglial modulation	[122]
Cerebral Endothelial Cells	Cerebral endothelial cells (CEC)	CEC‐derived sEVs	BV2 and primary microglia ± LPS; Transwell uptake assays; NF‐κB, TAB2–TAK1, autophagy readouts	Repeated LPS‐induced neuroinflammation in mice	Neuroinflammation associated with NDDs	Shifted microglia to anti‐inflammatory state ↓ pro‐inflammatory cytokines (IL‐6, IL‐1β, TNF‐α) Enhanced autophagy ↓NLRP3 Improved cognition and affective behavior in LPS‐treated mice	Yes	LPS‐based inflammation model (not NDD‐specific); male mice only; EV heterogeneity not dissected; long‐term safety not assessed	[124]
Oligodendrocytes	Mouse primary oligodendrocyte	Oligodendrocytes‐derived sEVs	Proteomic and lipidomic analyses	NA	Neural Protection	sEVs enriched in myelin proteins + protective enzymes; proposed to support axon–glia communication and myelin maintenance	Yes	Foundational study; no disease or functional in vivo validation	[87]

In a nutshell, these studies highlight the intrinsic ability of healthy neural cell–derived sEVs to mitigate neural insults associated with NDDs by modulating inflammation, supporting neuronal survival, and promoting repair mechanisms. Ongoing efforts to isolate and characterize sEVs from specific neural cell types, including neurons, astrocytes, microglia, oligodendrocytes, and neural stem cells, are paving the way for the development of targeted sEV‐based therapies. Whether used in their native form or modified to incorporate therapeutic agents, neural sEVs offer a versatile platform for drug delivery in NDDs. Given their established role as DDS [128, 129, 130] and broad coverage in recent reviews [131, 132, 133], neural sEVs represent a promising strategy for delivering neuroprotective and disease‐modifying agents. Their innate organotropism toward the brain further enhances their value by potentially facilitating drug passage across the BBB and choroid plexus (CP), offering a targeted and minimally invasive approach to treating neurodegenerative diseases. However, the predominance of short‐term, single‐model studies, variability in donor cell state, and limited standardization across isolation and dosing paradigms, collectively constrain translational readiness. Addressing these limitations through comparative, longitudinal, and mechanistically resolved studies will be essential to fully harness neural sEVs as therapeutic and drug‐delivery platforms in neurodegenerative diseases.

## 4 sEVs as Drug Delivery System in NDDs

Current FDA approved treatments for NDDs predominantly emphasize symptomatic management, aiming to mitigate the effects of neurotransmitter disturbances, rather than directly addressing the underlying pathogenic pathways. As a result, these treatments fall short of offering a cure or significantly delaying the development and progression of these diseases [134]. A major barrier to developing effective therapies is the BBB, whose selective permeability, tight junctions, and efflux transporters severely restrict the entry of most drug molecules. This highlights the critical importance of innovative biomaterial‐based DDS that can safely and efficiently deliver therapeutics to the brain, overcoming BBB‐associated challenges [22].

### 4.1 Barriers to the Drug Delivery to Brain

The brain is protected from the periphery by different barriers, including the BBB and the choroid plexus (CP). The BBB is primarily composed of CECs, basement membrane, pericytes, astrocyte end feet, and glial podocytes [135]. It is a complex network in the CNS that helps protect the brain and maintain its homeostasis. It allows limited transcytosis and conditional permeability, only becoming disrupted under certain pathological conditions [136]. The BBB uses selective cells and structures to control the entry of nutrients and prevent toxins from entering the brain [35, 36]. Molecules that do cross by passive diffusion must meet strict criteria, such as molecular weight below 400 Da and a non‐ionized state [34]. Consequently, many small‐molecule therapeutics cannot traverse the BBB unaided, making drug delivery to the brain a formidable challenge. sEVs have recently emerged as promising biologically compatible carriers capable of bypassing these restrictions, although the precise mechanisms by which they cross the BBB remain under active investigation [137].

The CP on the other hand is a cauliflower‐like structure projecting into the four ventricles, which provides an additional interface for CNS exchange. It is responsible for producing CSF, supplying nutrients and hormones, and clearing waste and toxins. Recent evidence suggests that, alongside the BBB, the CP may serve as a valuable target for CNS drug delivery by exploiting the blood–CSF barrier [138, 139]. This barrier is formed by cuboidal choroid plexus epithelial (CPE) cells, whose apical tight junctions restrict entry into the CSF, while their basal surface faces fenestrated blood vessels that permit free molecular exchange [140]. The barrier's functional capacity is further amplified by extensive basolateral infoldings and a dense apical microvilli network, giving it a large effective surface area which, in rats, is estimated to be nearly half that of the BBB [141]. Importantly, the blood–CSF barrier differs from the BBB in distributional outcomes. While the BBB penetration enables widespread brain delivery, CP targeting primarily favors deposition into the CSF and adjacent brain–CSF contact zones [142]. In the context of sEV‐based delivery, this distinction is particularly relevant, as sEVs that traverse the blood–CSF barrier are more likely to distribute via the CSF and brain–CSF interfaces, whereas sEVs crossing the BBB can achieve broader parenchymal penetration.

### 4.2 sEVs Circumventing the Barriers

**FIGURE 5:**
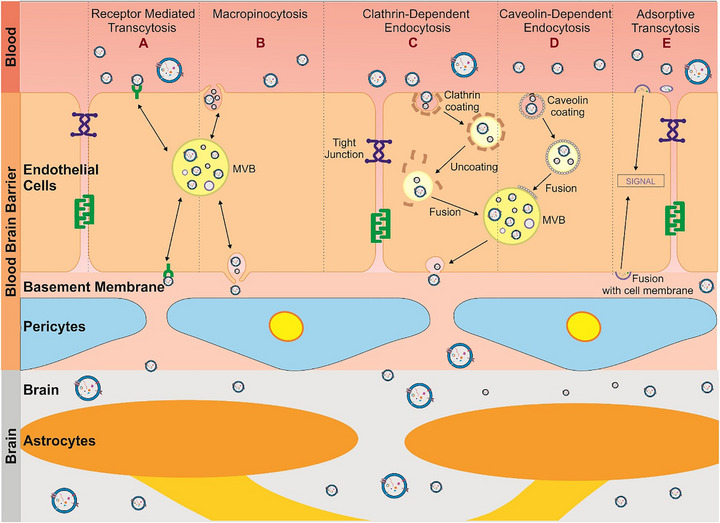
sEV transport pathways across the blood‐brain barrier. (A) Receptor‐mediated transcytosis: sEVs bind to specific receptors on endothelial cells and are internalized into vesicles that traverse the cell before release into the brain parenchyma. (B) Macropinocytosis: sEVs are engulfed in large, non‐selective endocytic vesicles formed by plasma membrane ruffling and internalized into multivesicular bodies (MVBs). (C) Clathrin‐dependent endocytosis: sEVs are taken up via clathrin‐coated pits, which undergo vesicle scission, uncoating, and fusion with endosomes or MVBs before transcytosis. (D) Caveolin‐dependent endocytosis: sEVs are internalized through caveolin‐coated vesicles, which fuse with MVBs and are subsequently transported across endothelial cells. (E) Adsorptive transcytosis: electrostatic interactions between positively charged sEVs and negatively charged endothelial surfaces trigger internalization and transcellular transport, followed by fusion with the abluminal membrane for release into the brain.

Once secreted, sEVs circulate in body fluids, such as blood and CSF, where they interact with recipient cells either by engaging surface receptors to trigger signaling cascades or by delivering cargo through internalization and membrane fusion [143]. Importantly, sEVs can cross the BBB bidirectionally, although the precise transport routes and their effects on ECs remain unclear [79]. Several receptor–ligand interactions have been implicated, including LFA‐1/ICAM‐1 [144], transferrin/transferrin receptor [145], CD46 [146] and the mannose receptor [147]. In addition, multiple transport mechanisms have been identified, such as clathrin‐ and caveolin‐dependent endocytosis [144], macropinocytosis [144] and adsorptive transcytosis [145, 147, 148], as illustrated in Figure 5. Banks et al. further demonstrated the ability of sEVs to cross the BBB using ten different EV preparations from human and murine cell lines. Although all samples successfully traversed the barrier, their transport rates varied and were strongly influenced by inflammatory conditions, such as lipopolysaccharide exposure [147]. Together, these findings emphasize the intrinsic organotropism of sEVs and reinforce their potential as natural DDS by leveraging their innate capacity for targeted tissue delivery.

### 4.3 Organotropism in sEVs

Organotropism refers to the ability of sEVs to preferentially target tissues that correspond to their cellular origin. Several studies have shown that systemically administered sEVs display a homing effect toward their source tissue, a phenomenon most evident in tumor‐derived vesicles. This property is largely attributed to the tumor‐specific integrin expression profiles present on their surfaces, which confer efficient organotropism and form the basis for exploiting these vesicles as advanced DDS [148, 149, 150]. Hoshino et al. demonstrated that sEV‐associated integrins dictate organ‐specific targeting, particularly to the lung, liver, and brain [151]. In line with this, integrin‐α2β expression on sEVs has been linked to brain metastasis, while integrin‐α4 has been associated with lymph node metastasis [152].

**FIGURE 6:**
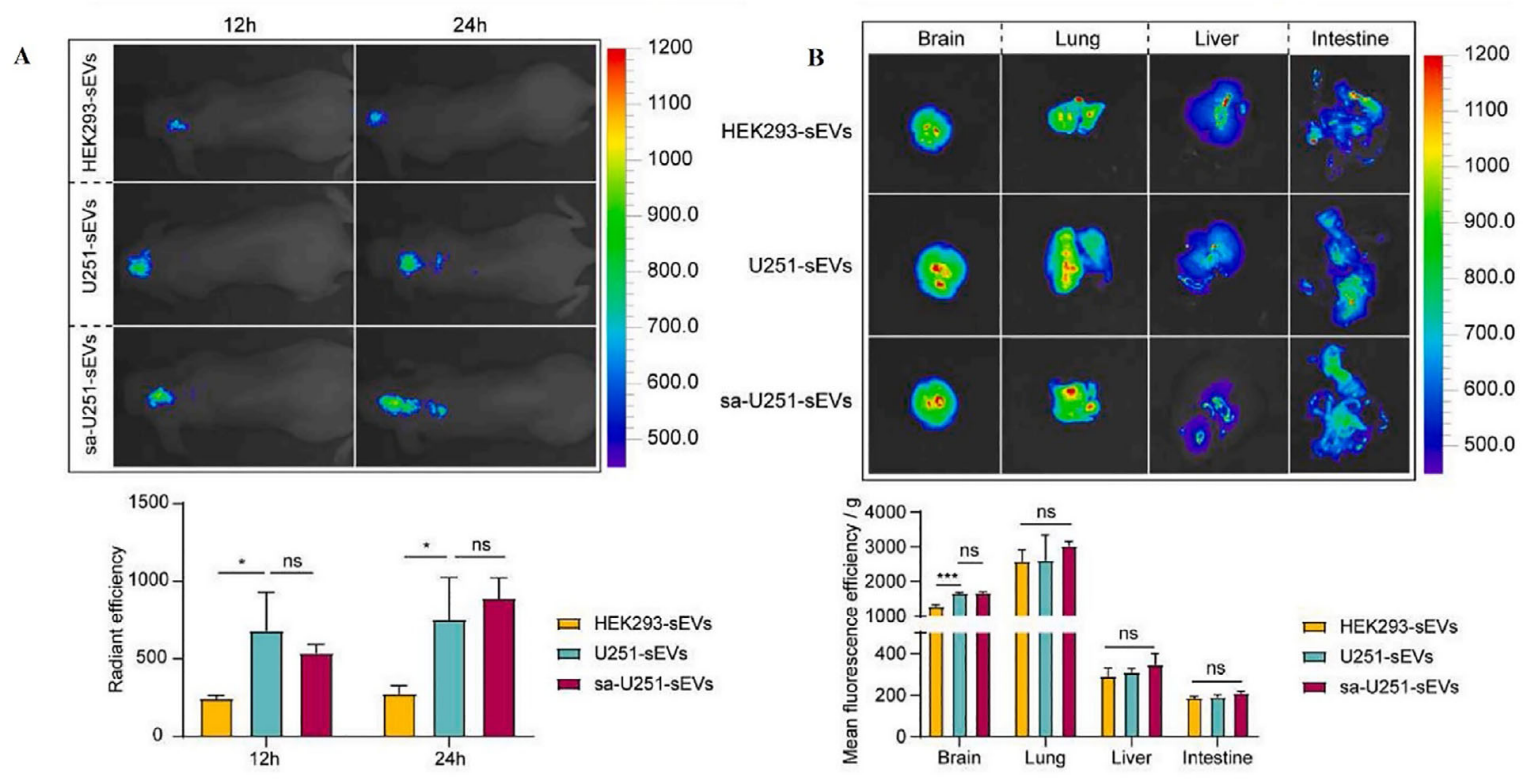
(A) In vivo fluorescence imaging of orthotopic brain tumor‐bearing mice using the IVIS Spectrum/CT system (top), with corresponding quantitative analysis of DiR fluorescence intensity (bottom). (B) Ex vivo fluorescence imaging of major organs collected 24 h after intravenous injection of DiR‐labeled sEVs (top), alongside statistical analysis of fluorescence distribution across organs (bottom). ^*^ *p <* 0.05, ^***^ *p <* 0.001, ns = not significant. HEK293‐sEVs (HEK293 cell‐derived sEVs as non‐GBM cell‐derived sEVs controls); U251‐sEVs (U251 cell‐derived sEVs as GBM cell‐derived EVs controls); sa‐U251‐sEV (saponin treated U251 cell‐derived sEVs). Reproduced under the terms of the Creative Commons CC‐BY license [154]. 2022 The Authors. Published by Elsevier B.V. on behalf of KeAi Communications Co., Ltd.

While these findings are promising, it is important to note that co‐purified cargo in the isolated sEVs may also be transmitted to recipient cells, potentially causing adverse effects. To mitigate this risk, safer and non‐immunogenic sources of sEVs are required [153]. Addressing this concern, Guo et al. developed a strategy to remove the cargo of glioblastoma (GBM)‐derived sEVs, while preserving their membranes, which retain the inherent targeting properties. These engineered sEVs exhibited an improved biosafety profile while maintaining the homing characteristics of native GBM‐derived vesicles [154]. They compared three different cargo elimination techniques and illustrated that the saponin treatment achieved the optimal cargo elimination, while also maintaining membrane integrity. Cellular uptake and flow cytometric analyses confirmed that saponin‐treated sEVs (S‐EVs) retained their inherent targeting efficiency (*p* < 0.001). In vivo analysis using an orthotopic brain tumor model of nude mice revealed the sEVs ability to penetrate the BBB (Figure 6A) and demonstrated brain homing abilities (*p <* 0.05). The accumulation concentration of S‐EVs in the lungs was slightly higher than in the brain; however, as compared to other major organs, the highest concentration was reached in the brain, as shown in Figure 6B.

Pauwels et al. also conducted a noteworthy study utilizing sEVs derived from immortalized CP epithelial cells (immCPE‐EVs), showcasing their homing capabilities toward the brain [155]. In their investigation, fluorescent tracing of sEV biodistribution following systemic administration of labeled sEVs indicated an augmented accumulation of immCPE‐EVs in the brain compared to the control (*p* < 0.001). The authors attributed this enhanced brain targeting to the presence of folate receptor 1 (FOLR1) in the CP. It is noteworthy that FOLR1 is also expressed in kidney epithelial cells, leading to a higher accumulation of immCPE‐EVs in the kidneys. Subsequent flow cytometric analysis revealed the highest uptake of immCPE‐sEVs by CP epithelial cells (*p* < 0.0001), suggesting the involvement of the blood‐CSF barrier in facilitating the transport of these sEVs into the brain. A functional cargo delivery study after intracerebroventricular injection of CRE recombinase enzyme‐loaded immCPE sEVs showed CRE delivery to microglia and astrocytes; however, neuronal uptake was not observed, showing a targeted cell delivery capability. This study is significant since these sEVs are derived from non‐tumor cells, highlighting their potential use as a targeted delivery vehicle in neuro‐nanomedicine owing to their low immunogenicity.

## 5 Bioengineering Strategies to Enhance Neural sEV Therapeutics

sEVs are increasingly recognized as promising vehicles for the delivery of therapeutic agents to specific cells and tissues. Their surface expression of adhesive and targeting proteins, such as LFA1/ICAM1, enables efficient interaction with recipient cells and facilitates selective uptake [156]. Importantly, multiple studies have demonstrated that sEVs can transport small molecules across the BBB and alleviate pathological features in CNS disorders, including AD [104] PD [157], ALS [158] and brain cancer [159]. Compared with synthetic DDS, sEVs offer distinct advantages, including natural biocompatibility, stability, intrinsic permeability across biological barriers, and organotropic homing capabilities, making them highly attractive for precision drug delivery.

Despite differences in size and cellular origin, sEVs share conserved mechanisms for cargo transport [160]. Their native cargo, comprising proteins, lipids, mRNAs, and miRNAs, plays important roles in tissue repair, neurogenesis, angiogenesis, and functional recovery, further underscoring their therapeutic relevance [143, 144, 145, 161]. Given the pivotal roles of sEV cargo, substantial effort has turned toward bioengineering sEVs (both natural and synthetic variants) to improve their therapeutic potential for AD and PD. Key strategies include altering sEV surfaces for targeting, loading sEVs with specific therapeutic cargo, and optimizing their stability and delivery. Below, we detail these approaches and highlight evidence of their efficacy.

### 5.1 Surface Engineering and Functionalization of sEVs

One strategy to enhance the therapeutic performance of sEVs is surface engineering with targeting ligands or stealth coatings to improve cell‐specific delivery and functional efficacy. This can be achieved either by genetic modification of parent cells to express vesicle surface proteins fused to targeting peptides, or by chemical conjugation of targeting moieties onto purified sEV membranes [162, 163]. Commonly used scaffold proteins, such as Lamp2b or lactadherin, have been fused to peptides that bind specific brain cell receptors. For example, microglial cells engineered to secrete sEVs displaying lactadherin (which binds phagocyte receptors) together with the anti‐inflammatory cytokine IL‐4 produced dual‐functional vesicles that showed enhanced uptake by brain phagocytes (*p* < 0.001) and promoted a therapeutic M2 microglial phenotype in vitro, demonstrating improved target engagement through surface display engineering [164]. Similarly, sEVs decorated with platelet‐derived growth factor (PDGF‐AA) have been generated by overexpressing PDGF‐A in donor neural cells, resulting in vesicles that selectively homed to PDGF receptors on oligodendrocytes in a demyelination model. When loaded with the reparative hormone triiodothyronine (T3), these sEVs successfully delivered cargo to oligodendrocytes, promoting remyelination and lesion improvement, thereby illustrating the synergy between surface targeting and therapeutic cargo loading [165].

Chemical surface functionalization provides an alternative and versatile strategy. Approaches such as click chemistry or maleimide–thiol coupling enable the attachment of peptides, antibodies, or polymers to sEV membranes, without compromising vesicle integrity. For instance, anchoring a hyaluronic acid (HA) derivative onto sEV surfaces enabled targeting of CD44‐expressing cells in a tumor model, generating pH‐responsive, cell‐specific vesicles [129]. Analogous strategies could be adapted for AD and PD, such as functionalizing sEVs with ligands targeting transferrin or insulin receptors to enhance BBB transport and neuronal uptake. While sEVs inherently exhibit some degree of brain tropism and BBB permeability [154], surface engineering seeks to further increase targeting specificity and reduce off‐target clearance.

Beyond direct modification, hybrid nanovesicles formed by fusing natural sEVs with synthetic liposomes or polymers represent an emerging approach, combining the biocompatibility and targeting properties of sEVs with the enhanced stability and circulation control of synthetic nanocarriers [166]. Collectively, these strategies demonstrate how surface engineering can be used to tailor neural sEVs for selective recognition of brain cell types or pathological niches in AD and PD, thereby improving localization to disease‐relevant sites while minimizing uptake by off‐target organs such as the liver and spleen.

### 5.2 Loading Therapeutic Cargo into sEVs

Efforts have been made to develop strategies to enhance therapeutic loading and delivery efficiency of therapeutic goods using sEVs. Broadly, two main approaches are employed for loading therapeutics into sEVs: endogenous and exogenous loading [167].

#### 5.2.1 Endogenous Loading Strategies

**FIGURE 7:**
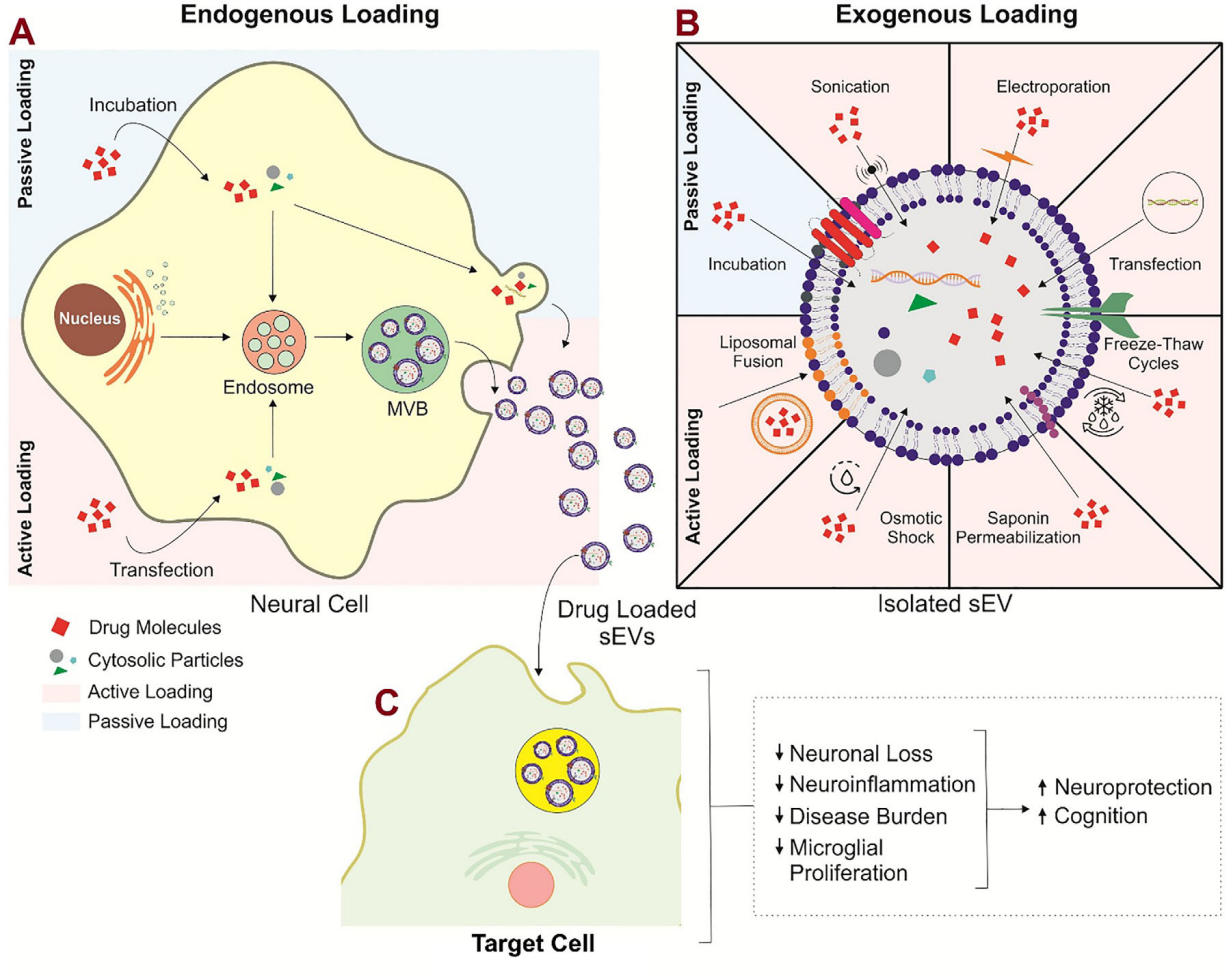
Graphic illustration of cargo loading in sEVs. (A) Endogenous methods: Passive loading is achieved by incubating donor cells with drug molecules, allowing spontaneous incorporation into secreted sEVs, while active loading uses transfection to drive cellular machinery to package therapeutic cargo into sEVs. (B) Exogenous methods: Passive loading involves simple incubation of isolated sEVs with drugs, whereas active loading employs techniques such as sonication (membrane disruption by ultrasound), electroporation (temporary pore formation with electric pulses), transfection (genetic modification of sEVs), freeze–thaw cycles (membrane destabilization and resealing), saponin permeabilization (membrane pore formation), osmotic shock (rapid osmotic changes for drug entry), and liposomal fusion (direct fusion of drug‐loaded liposomes with sEV membranes). (C) Reported effects of drug loaded Neural cell‐derived sEVs on target cells.

Endogenous loading relies on modifying donor cells so that therapeutic cargo is naturally incorporated into sEVs during biogenesis (Figure 7A). In this approach, cells are incubated with or transfected by genetic material, small molecules, pharmaceuticals, or nutraceuticals, which subsequently enter the endosomal or plasma membrane pathways and become encapsulated within sEVs through the cell's endogenous sorting machinery. The resulting vesicles are released as exosomes or ectosomes. Although advances have been made in understanding intracellular trafficking pathways, the precise molecular mechanisms governing selective cargo incorporation into sEVs remain incompletely understood [131, 168, 169].

Cell selection is a critical consideration for endogenous loading, as donor cells expressing surface proteins and adhesion molecules compatible with target tissues can enhance the targeting specificity of the resulting sEVs [169]. Endogenous approaches are particularly well suited for biological cargos such as miRNAs, siRNAs, and therapeutic proteins, as they exploit physiological loading pathways and preserve vesicle integrity. However, this strategy is generally less suitable for the reproducible encapsulation of chemically synthesized small‐molecule drugs and often yields relatively low cargo loading efficiency [170].

Beyond simple cargo enrichment, endogenous loading can be extended through genetic bioengineering of donor cells. Neural cells, macrophages, and microglia have been engineered to overexpress therapeutic proteins, neurotrophic factors, or regulatory RNAs, resulting in sEVs enriched with disease‐modifying cargo. More sophisticated designs have demonstrated the feasibility of packaging multiple therapeutic components within the same sEV, enabling combinatorial interventions targeting inflammation, protein aggregation, and neuronal survival simultaneously.

#### 5.2.2 Exogenous Loading Strategies

Exogenous loading involves the incorporation of therapeutic agents into purified sEVs after isolation. This strategy can be subdivided into passive and active methods. Passive loading typically involves incubating sEVs with lipophilic drugs, allowing association with the vesicle membrane, as demonstrated by Yang et al., who successfully loaded paclitaxel and doxorubicin into sEVs through simple incubation [149]. While gentle, this approach is often limited by low loading efficiency and is primarily applicable to hydrophobic compounds.

Active exogenous loading techniques transiently disrupt the sEV membrane to facilitate cargo entry. Commonly used methods include electroporation [166, 171], sonication [172], transfection [173], freeze thaw cycles [174], saponin permeabilization [175], osmotic shock [175], liposomal fusion [175] and mechanical extrusion [157], as illustrated in Figure 7B. These approaches generally achieve higher loading efficiencies than endogenous strategies [21, 157], particularly for small RNAs and small‐molecule drugs. However, the mechanical and chemical stresses involved may compromise vesicle integrity, stability, and bioactivity if not carefully optimized [161]. In addition, drug physicochemical properties, vesicle lipid composition, and intrinsic molecular cargo can all influence loading efficiency and post‐loading performance [21].

For example, in the PDGF‐A–decorated sEV system described above, therapeutic loading was achieved by sonicating sEVs in a solution containing the thyroid hormone triiodothyronine (T3), enabling efficient encapsulation of the drug [165]. Electroporation has likewise been widely used to load nucleic acids into sEVs for neurodegenerative disease therapy. A seminal study by Alvarez‐Erviti et al. demonstrated that electroporated siRNA targeting BACE1, delivered via targeted sEVs, reduced cortical BACE1 expression and amyloid‐β levels in wild‐type C57BL/6 mice, representing one of the earliest proofs of concept for sEV‐mediated brain drug delivery [176]. In another study, dopamine was encapsulated into blood‐derived sEVs and combined with rabies virus glycoprotein (RVG) peptide for neuronal targeting. Systemic administration of these dopamine‐loaded sEVs enabled BBB crossing and significantly improved motor functions in 6‐OHDA PD model mice (p<0.05–0.001) [145].

Each loading strategy presents distinct advantages and limitations. Electroporation is effective for RNA delivery, but may cause cargo leakage or vesicle aggregation if not carefully optimized. Sonication and extrusion achieve higher loading efficiencies for small‐molecule drugs, but can disrupt membrane integrity or alter protein orientation, whereas passive incubation is gentler, yet often results in low encapsulation efficiency, unless the cargo is lipophilic [177]. Emerging approaches, such as vesicle mimetics, generated by mechanically disrupting cells or vesicles and reforming them in the presence of therapeutic cargo (e.g., serial extrusion through microscale filters), offer higher production yields and improved scalability while enabling cargo incorporation during fabrication [177, 178]. Collectively, the choice of loading strategy depends on cargo properties (e.g., hydrophilic vs. hydrophobic, nucleic acid vs. protein) and the need to preserve vesicle functionality post‐loading.

### 5.3 Enhancing sEV Stability and Delivery Efficiency

Stability during storage and circulation represents another major challenge for sEV‐based therapeutics. Native sEVs possess lipid membranes enriched in cholesterol and tetraspanins, conferring relative stability in vivo, and are less rapidly cleared than synthetic liposomes, due in part to the expression of “self” markers, such as CD47 [179]. Nevertheless, aggregation and loss of bioactivity can occur during long‐term storage or repeated freeze–thaw cycles. Bioengineering strategies to address these issues include PEGylation, which reduces aggregation and immune recognition, and lyophilization with cryoprotectants to enable long‐term dry storage [180]. For instance, incorporation of trehalose during freeze‐drying has been shown to preserve sEV size and functional integrity upon rehydration [180].

Efficient brain delivery remains a central hurdle, as crossing the BBB and achieving sufficient parenchymal penetration are limiting factors for most therapeutics. sEVs offer inherent advantages in this regard, owing to their size and surface proteins that can engage endothelial receptors to facilitate transcytosis [181]. In vivo studies have demonstrated that peripherally administered sEVs can reach the brain parenchyma, particularly with repeated dosing [110]. Delivery efficiency can be further enhanced through surface modification with targeting ligands, such as RVG peptides to exploit receptor‐mediated transport [182], or peptides that promote extracellular matrix interactions and tissue distribution [183]. Alternative administration routes, including intranasal delivery, have also shown promise by bypassing the BBB via olfactory and trigeminal pathways; intranasally delivered MSC‐ or NSC‐derived sEVs have improved cognitive outcomes in AD models, consistent with direct nose‐to‐brain transport [184]. In parallel, strategies to reduce rapid clearance, such as PEGylation or optimized dosing regimens, can prolong circulation time and maximize brain uptake [185]. Overall, advances in stabilizing sEV formulations and enhancing brain delivery are critical to fully harness their natural biocompatibility and BBB‐crossing potential for therapeutic applications.

### 5.4 Improving Therapeutic Efficacy: Preclinical Evidence

Bioengineered neural sEVs have demonstrated promising therapeutic efficacy in preclinical models of AD and PD, in many cases outperforming unmodified sEVs or conventional drug formulations. A landmark example is the study by Alvarez‐Erviti et al., in which RVG‐targeted sEVs were used to deliver BACE1 siRNA, resulting in approximately 50% reduction of brain amyloid‐β levels in C57BL/6 mice, a direct disease‐modifying effect relevant to AD [176]. More recently, Ge et al. (2020) loaded miR‐124 into microglial sEVs and showed that systemic administration enhanced Aβ clearance and improved memory in APP/PS1 mice, consistent with the ability of miR‐124 to modulate inflammatory pathways and promote microglial phagocytosis [77]. Together, these studies illustrate the capacity of engineered sEVs to deliver gene‐regulatory cargos and modulate core AD pathology.

Therapeutic benefits have also been reported in PD models. In the GDNF‐sEV macrophage study by Zhao et al., treated Parkin Q311X(A) mice exhibited significantly increased nigral dopamine levels and improved motor performance compared with controls, indicating neurorestorative effects in dopaminergic pathways (*p* < 0.05–0.001) [186]. In a related approach, neural progenitor cell–derived sEVs loaded with neuroprotective peptides such as NR2B9c, reduced infarct size and improved neurological scores in a controlled cortical impact mouse stroke model [187], highlighting broader relevance to neuroprotection and pathways intersecting with PD pathophysiology.

An additional and highly translational application involves using sEVs to deliver conventional drugs with limited brain penetration. Qu et al. (2018) encapsulated dopamine within blood plasma–derived sEVs and demonstrated BBB crossing and significant alleviation of parkinsonian symptoms in 6‐OHDA mouse PD model, with higher brain dopamine levels and reduced peripheral toxicity compared with free dopamine [145]. Similarly, neuron‐derived sEVs loaded with anti‐inflammatory compounds, such as curcumin, showed superior suppression of neuroinflammation in an LPS‐induced brain inflammation model relative to free drug, attributable to improved microglial delivery [188]. Parallel successes in oncology, where sEVs loaded with chemotherapeutics, such as paclitaxel, effectively suppressed brain metastases, further reinforce the general principle that sEV encapsulation enhances CNS bioavailability and targeting, often allowing therapeutic efficacy at lower doses [150, 172].

Together, these studies highlight bioengineered neural sEVs as versatile platforms for delivering nucleic acids, peptides, and small‐molecule drugs to the CNS with enhanced efficacy. Although therapeutic outcomes remain model‐ and context‐dependent, accumulating preclinical evidence demonstrates their potential to alleviate neuroinflammation, restore mitochondrial function, enhance synaptic plasticity, and reduce pathological protein accumulation, as summarized in Figure 7C. Collectively, these findings support the translational potential of engineered sEVs as adaptable disease‐modifying strategies for neurodegenerative disorders.

### 5.5 Quality Control and Analytical Considerations for Engineered sEVs

As engineered sEVs advance toward translational and clinical applications, robust quality control (QC) and analytical frameworks become essential to ensure reproducibility, safety, and therapeutic reliability. Whereas basic characterization may be sufficient for exploratory studies, engineered sEV formulations require additional layers of validation to confirm vesicle identity, purity, and functional potency [189, 190]. Identity assessments typically include verification of vesicle size distribution, morphology, and enrichment of EV‐associated markers, alongside confirmation of successful cargo incorporation or surface modification [190].

Purity considerations are particularly critical for engineered sEVs, as loading procedures and post‐isolation modifications may introduce non‐vesicular contaminants, residual reagents, or structurally compromised vesicles [191, 192]. Comprehensive evaluation of co‐isolated proteins, lipoproteins, and other nanoparticles is therefore necessary to ensure that observed biological effects can be attributed specifically to engineered sEVs rather than to co‐purified components [192, 193]. Potency assays represent an additional and often underdeveloped dimension of sEV analytics. Functional readouts aligned with the intended mechanism of action, such as target engagement, modulation of inflammatory pathways, or neuroprotective effects, are increasingly recognized as essential complements to physicochemical characterization [194, 195].

Beyond these core metrics, preparing engineered sEVs for clinical development requires early consideration of manufacturing readiness. During process scale‐up, it is important to define key product attributes such as size consistency, cargo integrity, and biological activity, and to monitor these parameters throughout production. Implementing a structured “quality by design” approach [196] during development can help identify potential sources of variability early and improve batch‐to‐batch consistency. In practical terms, monitoring upstream parameters (e.g., cell health and culture conditions) and downstream purification steps (e.g., filtration or chromatography performance) can help detect deviations before final product release. Establishing clear quality benchmarks early in development reduces the risk of late‐stage manufacturing failures and facilitates a smoother transition from laboratory‐scale production to larger, GMP‐compliant systems [196, 197].

Collectively, the integration of identity, purity, potency, and manufacturing‐oriented quality controls will be essential to ensure that engineered sEVs can progress from promising preclinical platforms to scalable and clinically viable therapeutics.

### 5.6 Design Decision Considerations for Engineering sEVs

Beyond analytical validation, the translational success of engineered sEVs is strongly influenced by upstream design decisions that govern manufacturability, stability, immunogenicity, and regulatory complexity. Genetic display approaches, achieved through modification of parent cells, offer high reproducibility and molecular precision, enabling stable incorporation of targeting ligands or therapeutic cargos [198, 199]. However, these strategies may introduce additional regulatory burden due to permanent genetic modification and can complicate large‐scale manufacturing and batch‐to‐batch consistency.

Chemical conjugation strategies provide greater post‐isolation flexibility and avoid genetic manipulation, potentially reducing regulatory risk and enabling modular formulation design. Nevertheless, these approaches may affect vesicle integrity, introduce residual reagents, and generate heterogeneous surface modifications, with implications for stability and immunogenicity [200]. Hybrid strategies, which combine genetically encoded features with post‐isolation chemical loading or conjugation, aim to balance these trade‐offs by integrating targeting specificity with formulation adaptability, though they often increase analytical and QC demands [20, 201].

Viewed collectively, these considerations form a practical design decision framework in which the choice of engineering strategy should be guided not only by biological performance but also by scalability, stability, safety, and regulatory feasibility. Aligning engineering design with manufacturing and quality requirements at an early stage can help avoid translational bottlenecks and improve the likelihood of successful clinical development.

## 6 Engineering Neural Cell‐Derived sEVs for Therapeutic Delivery to the Brain

Building on the therapeutic promise of sEVs discussed earlier, including their biocompatibility, organotropism, and ability to cross brain barriers, sEVs derived from neural cells have emerged as particularly attractive candidates for delivering therapeutics in NDDs. A growing body of literature demonstrates the feasibility of engineering neural cell‐derived sEVs to encapsulate and deliver a variety of bioactive cargos, including proteins, RNAs, and small molecules, directly to the brain. However, most studies remain proof‐of‐concept in nature, relying on preclinical models and short‐term outcome measures, with limited evaluation of dosing reproducibility, long‐term safety, or scalability.

### 6.1 Astrocytes‐Derived sEVs

As previously discussed, astrocyte‐derived sEVs possess intrinsic neuroprotective and disease‐modulating properties. However, strategic modifications to these sEVs can further enhance their therapeutic potential. Pascua–Maestro et al. utilized Lipocalin ApoD‐positive astrocyte cell lines to isolate sEVs, capitalizing on the neuroprotective properties of Lipocalin ApoD. This protein, exclusively transferred via sEVs from astrocytes to neurons, regulates lipid peroxide levels generated by reactive oxygen species (ROS) accumulation. Using an ApoD‐knock‐out (KO) mouse model, the study demonstrated successful ApoD uptake by neurons under oxidative stress (*p <* 0.01), promoting their functional integrity and survival [69]. While mechanistically informative, this approach relies on genetically defined models and does not address whether ApoD enrichment can be consistently achieved under clinically scalable conditions. In a study conducted by Liao et al., long intergenic non‐coding RNA (lincRNA)‐Cox2‐siRNA was effectively loaded into sEVs derived from primary astrocytes through transfection and delivered to the brain via the intranasal route of administration. LincRNA‐Cox2 plays a crucial role in controlling the expression of a subset of cell cycle genes involved in lipopolysaccharide (LPS)‐induced microglial cell proliferation, a common occurrence in NDDs. Its downregulation has been shown to reverse LPS‐induced microglial proliferation, thereby inducing neuroprotective effects in NDDs. Using mouse primary astrocyte‐derived sEVs loaded with lincRNA‐Cox2‐siRNA, Liao and colleagues demonstrated the reversal of LPS‐induced microglial proliferation in both in vitro and in vivo models (*p <* 0.001). The study revealed that sEVs carrying lincRNA‐Cox2‐siRNA were effectively taken up by microglial cells in vitro and reached the brain in vivo via the intranasal route of administration [184]. As mentioned earlier, while intranasal delivery offers translational appeal, variability in nasal‐to‐brain delivery efficiency and reliance on acute inflammatory models may limit direct extrapolation to chronic NDD settings.

In another study, Peng et al. examined astrocyte‐derived sEVs modified by treatment with acidic fibroblast growth factor (haFGF14‐154) in AD models. In vitro, these sEVs promoted neurite growth, enhanced synaptic protein expression (PSD95, GAP43, synaptophysin), and reduced Aβ burden in Aβ‐injured cortical neurons (*p* < 0.05‐0.0001). Intranasal administration of modified sEVs in APP/PS1 and Aβ‐injected mice improved spatial memory and synaptic ultrastructure, while decreasing plaque accumulation. Mechanistically, their effects were mediated by downregulation of miR‐206‐3p, which saw a >4 fold upregulation in disease conditions, however was restored back to normal after treatment, which in turn restored brain‐derived neurotrophic factor (BDNF), inhibited δ‐secretase, and attenuated AD pathology [202]. Despite the robust molecular characterization done in this study, the dependence on a single regulatory miRNA pathway raises questions regarding durability and off‐target effects in heterogeneous disease contexts. Wen et al. investigated whether sEVs derived from fibroblast growth factor‐2 (FGF2)‐primed astrocytes could alleviate mitochondrial and synaptic toxicities associated with PD. In vitro, they reversed MPP+‐induced mitochondrial dysfunction, reduced ROS, and restored synaptic protein levels (*p* < 0.01–0.0001). In vivo, stereotactic administration into MPTP‐treated mice improved dopaminergic neuron survival, increased NCAM1 expression, and improved motor function (*p* < 0.05–0.001). Proteomic analysis revealed NCAM1 enrichment in FGF2‐sEVs, and siRNA knockdown of NCAM1 abolished their protective effects, establishing it as a key mediator of mitochondrial protection and synaptic restoration. Collectively, these findings highlight FGF2‐primed astrocyte‐derived EVs as a promising therapeutic strategy for PD by targeting both mitochondrial dysfunction and synaptic deficits [203]. However, the requirement for intracranial administration and growth factor priming introduces translational and regulatory complexities that warrant further consideration.

### 6.2 Microglia‐Derived sEVs

As previously noted, microglia‐derived sEVs inherently contribute to intercellular communication and immune modulation. However, by engineering these sEVs, their therapeutic potential can be significantly enhanced. Ge and colleagues demonstrated the potential of sEVs expressing miR‐124‐3p, derived from microglia, in neuroprotection and Aβ clearance in AD. miR‐124‐3p is a highly expressed miRNA in neural cells and is a key inflammation and immunity modulator. Using an in vitro repetitive scratch cell injury model, it was revealed that sEVs with upregulated miR‐124‐3p helped in alleviation of neuronal damage by suppression of Rela, which is an ApoE inhibitory transcription factor responsible for β‐amyloid cleavage (*p* < 0.001). Systemic tail vein injection in a rmTBI AD mouse model showed an increased uptake of miR‐124‐3p upregulated sEVs by neurons, resulting in more than 4‐fold increased miR‐124‐3p levels in the injured brain, where they provided neuroprotective effects. This was associated with overall improved cognition in mice [77]. Nevertheless, how this might translate to models of neurodegeneration has not yet been investigated.

Li et al. investigated the role of microglia‐derived sEVs in modulating neuroinflammation in Parkinson's disease. The study demonstrated that oligomeric α‐synuclein activated microglia through the PRAK/MK5 pathway, while monomeric α‐synuclein induced an anti‐inflammatory phenotype. sEVs released from monomeric α‐synuclein‐treated microglia (EVs+) were enriched with specific miRNAs, including miR‐466b‐3p, miR‐466c‐3p, and miR‐669c‐5p, which directly targeted PRAK. In vitro, EVs+ suppressed pro‐inflammatory mediators and enhanced anti‐inflammatory markers in microglia. In vivo, intravenously administered EVs+ successfully crossed the BBB and reprogrammed microglia toward an anti‐inflammatory phenotype in LPS‐challenged mice (*p* < 0.01–0.0001). These findings identify EV‐associated miRNAs as critical mediators of microglial polarization and suggest that targeting the PRAK pathway via sEV delivery represents a promising therapeutic strategy for alleviating PD‐related neuroinflammation [204]. Although mechanistically elegant, the stability of this engineered anti‐inflammatory phenotype over a prolonged disease course, however, still remains unresolved. Filannino et al. evaluated the therapeutic potential of sEVs loaded with chrysin (sEV‐C), a natural flavonoid with anti‐inflammatory properties, in modulating microglial activation. Using BV2 microglia stimulated with LPS, the authors demonstrated that sEV‐C significantly attenuated neuroinflammatory responses (*p* < 0.001). Treatment with sEV‐C reduced LPS‐induced proliferation, restored resting microglial morphology, and suppressed migratory capacity. Moreover, sEV‐C downregulated key pro‐inflammatory mediators, including IL‐1β, IL‐6, and caspase‐1, while upregulating the anti‐apoptotic protein Bcl‐xL, thereby promoting a shift toward an anti‐inflammatory, neuroprotective microglial phenotype (*p* < 0.001). Unlike free chrysin, sEV‐mediated delivery enhanced cellular uptake and biological efficacy, underscoring the promise of EVs as bio‐carriers for natural compounds in treating neuroinflammation and neurodegenerative disorders [205]. Nevertheless, reliance on BV2 cells and acute inflammatory stimulation limits insight into efficacy within complex in vivo neurodegenerative environments. An overview of reported neural cell–derived sEV‐based drug delivery strategies, including cargo types, loading approaches, experimental models, and therapeutic outcomes, is summarized in Table 2.

**TABLE 2:** Summary of engineered neural cell‐derived sEVs for therapeutic delivery modalities in NDDs.

Cell Type	sEVs Source	Active Component loaded in sEVs	Loading Method Used	In vitro Model/Design used	In vivo Model/Design used	Target Disease/ pathways	Therapeutic Outcome	Key Stats Report‐ed?	Limitations	Ref
Astrocytes	Mouse primary astrocytes	Long intergenic non‐coding RNA (lincRNA)	Exogenous Transfection	LincRNA‐Cox2 knockdown in microglial cells	C57BL/6NWT mice intra‐nasal administration	LPS induced microglial proliferation in NDDs	↓ LPS‐induced microglial proliferation ↓ BrdU^+^/Ki67^+^ microglia ↓ neuronal loss	Yes	Acute LPS model; no NDD context; limited behavioral or long‐term outcome assessment	[184]
	human astrocytoma 1321N1, neuroblastoma SH‐SY5Y Cell lines and primary astrocytes	Apolipoprotein D positive sEVs	Endogenous Transfection	NA	ApoD‐knock‐out mice model	Oxidative stress associated with NDDs	Neuroprotection under oxidative stress ↓ Lipid peroxidation	Yes	Predominantly in vitro; no disease‐specific animal model; BBB delivery and dosing not assessed	[69]
	Rat primary astrocytes	sEVs from astrocytes treated with Aβ ± haFGF14‐154 (↓ miR‐206‐3p, ↑ BDNF)	Endogenous Incubation	Primary cortical neurons ± Aβ; synaptic protein assays	APP/PS1 AD Mouse Model	Alzheimer's disease	↓ Aβ accumulation, ↑ BDNF, ↓ δ‐secretase, improved cognition	Yes	Mouse AD model; scalability and reproducibility of sEV priming not addressed	[202]
	Mouse primary astrocytes	FGF2‐primed astrocyte sEVs enriched in NCAM1	Endogenous Incubation	MPP+ PD Model	MPTP PD mouse model	Parkinson's disease	Restored mitochondrial function, ↑ synaptic proteins, ↑ TH+ neurons, improved locomotion	Yes	Acute toxin model; limited relevance to chronic α‐syn pathology	[203]
Microglia	Mouse microglial cell line (BV2 cells)	sEVs loaded with flavonoid chrysin	Exogenous Incubation	Cell Proliferation Assay, Wound Healing Assay, Cytokine release	NA	Neuroinflammation related to Alzheimer's and Parkinsons disease	↓ IL‐1β, IL‐6, caspase‐1 restored resting phenotype	Yes	In vitro only; immortalized microglia may not reflect primary cells	[205]
	Mouse microglial cell line (BV2 cells) and Primary Microglia	sEVs from monomeric α‐synuclein‐treated microglia, enriched in miR‐466b‐3p, miR‐466c‐3p, miR‐669c‐5p	Endogenous Incubation	Cytokine release, Inflammasome assay	WT + SNCA‐KO mouse model	Parkinson's disease and neuroinflammation	Restored mitochondrial function, ↑ synaptic proteins, ↑ TH+ neurons, improved locomotion	Yes	Acute LPS model does not reflect progressive PD; long‐term effects not assessed	[204]
	Mouse microglial cell line (BV2 cells) transfected with miR‐124‐3p	miR‐123‐3p upregulated sEVs derived from transfected BV2 cells	Endogenous Transfection	Repetitive Scratch Injury Model	Repetitive mild traumatic brain injury (rmTBI) mouse model	Alzheimer's Disease associated rmTBI	↓ amyloid pathology, ↑ neurite outgrowth, normalized neurodegeneration markers (BDNF, neurogranin, VILIP‐1), ↑ learning and memory via the Rela/ApoE pathway	Yes	Complex injury model but limited dosing/PK data; sEV heterogeneity not fully resolved; long‐term safety and scalability not addressed	[77]
	Mouse microglial cell line (BV2 cells)	microRNA‐711 (miR‐711) encapsulated in microglia‐derived sEVs	Endogenous Transfection	BV2 microglia polarization and inflammatory assays	rmTBI mouse model using C57BL/6 mice	Alzheimer's Disease	↓ score of neurological deficits, ↓ Tau hyperphosphorylation via Itpkb inhibition, improved cognitive function and response	Yes	rmTBI rather than genetic AD model; single miRNA focus; long‐term durability and translational dosing not evaluated	[78]

Although research in this area is still emerging, current findings demonstrate that neural cell‐derived sEVs can be rationally engineered to deliver diverse therapeutic cargos across the BBB, resulting in modulation of neuroinflammation, mitochondrial function, synaptic integrity, and protein aggregation. However, the majority of approaches remain model‐specific, utilize heterogeneous loading strategies, and lack standardized comparisons across cell sources, cargos, and delivery routes. Addressing these limitations through head‐to‐head evaluations, longitudinal safety studies, and scalable manufacturing strategies will be essential for advancing engineered sEVs toward clinical translation in neurodegenerative diseases.

## 7 Current Challenges and Translational Pathways for sEV‐Based Therapies in NDDs

Despite their substantial promise as both DDS and direct therapeutic agents, several critical challenges continue to limit the clinical translation of sEV‐based therapies for NDDs. These barriers span scientific, manufacturing, regulatory, and logistical domains and must be addressed systematically to enable successful bench‐to‐bedside application.

From a biological and scientific perspective, the intrinsic heterogeneity and complex biochemical composition of sEVs pose major obstacles to standardization and reproducibility. Vesicle cargo varies significantly depending on donor cell type, cellular activation state, and culture conditions, complicating batch‐to‐batch consistency and therapeutic predictability. Moreover, specific protein or lipid components within sEVs may elicit immune recognition, leading to accelerated clearance and reduced efficacy [206, 207]. These challenges are further compounded by ongoing issues related to nomenclature, classification, and the precise definition of biologically relevant sEV subpopulations, which remain under active debate within the field [208, 209].

One of the most critical limitations affecting the interpretation of sEV‐based studies is the lack of methodological standardization in vesicle isolation and characterization [210]. Current studies employ diverse isolation techniques including ultracentrifugation, precipitation‐based methods, filtration, and chromatography, each yielding vesicle populations that differ in purity, size distribution, and cargo composition [210, 211]. Likewise, characterization practices vary widely, with inconsistencies in the reporting of vesicle size, surface markers (e.g., CD63, CD81), and membrane integrity [211]. Such variability can substantially influence downstream functional outcomes, including inflammatory responses, protein aggregation, and neuronal viability. Inadequate separation of sEVs from microvesicles or co‐isolated contaminants further complicates data interpretation [212]. While consensus frameworks, such as the Minimal Information for Studies of Extracellular Vesicles (MISEV) guidelines, represent an important step toward improving rigor and transparency, they primarily define minimal reporting requirements, rather than harmonized experimental protocols or functionally equivalent sEV populations [7]. As a result, substantial variability persists even among studies nominally adhering to MISEV, limiting cross‐study comparability and the feasibility of robust meta‐analyses.

Manufacturing and scalability represent some of the most significant translational hurdles. Current isolation techniques, including ultracentrifugation, often result in variable purity and co‐isolation of contaminants, limiting reproducibility and clinical‐grade production [180]. These challenges are exacerbated by the lack of upstream standardization, as scaling sEV production under Good Manufacturing Practice (GMP) conditions while maintaining consistent yield, cargo composition, and bioactivity remains technically demanding [213]. Neural stem cells (NSCs) and mesenchymal stem cells (MSCs) have emerged as promising donor sources, with MSC‐derived sEVs demonstrating relatively stable immunological tolerance profiles [214]. Emerging technologies, including bioreactor‐based culture systems and microfluidic platforms, offer potential solutions to improve yield, scalability, and standardization [215, 216], yet require further validation for clinical implementation.

Efficient and safe cargo loading presents an additional layer of complexity for clinical translation. Both endogenous and exogenous loading strategies can enhance therapeutic specificity, but aggressive post‐isolation manipulation may compromise vesicle integrity, alter biological activity, or induce donor‐cell stress [217]. Achieving an optimal balance between loading efficiency and preservation of native sEV functionality remains an unresolved challenge. Storage and distribution further complicate translation, as standardized preservation protocols are lacking. Although lyophilization has shown promise for cost‐effective long‐term storage at −20°C, additional studies are required to confirm its impact on vesicle stability and therapeutic efficacy [218].

Regulatory and logistical considerations are equally critical. As biological products, sEV‐based therapeutics must comply with stringent safety, efficacy, and manufacturing standards, yet universally accepted regulatory frameworks and clinical guidelines have not been fully established [219]. Reproducibility between batches, long‐term safety, biodistribution consistency, and optimized administration routes all represent essential criteria for regulatory approval [180]. While sEVs are generally considered biocompatible, immunogenicity concerns may arise, particularly for allogeneic, engineered, or hybrid vesicles. Although early clinical studies, primarily in oncology, suggest favorable tolerability [220], comprehensive long‐term safety assessments in neurological contexts remain necessary.

An additional challenge influencing interpretation and translation is potential publication bias in preclinical sEV studies, particularly those employing animal models of AD and PD. Selective reporting of positive or therapeutic outcomes, combined with heterogeneity in disease models and outcome measures, may inflate perceived efficacy and obscure model‐dependent limitations [221]. Greater transparency, including the publication of rigorously designed studies reporting neutral or negative outcomes, would improve reproducibility, reduce unnecessary duplication of effort, and support more rational experimental design.

Bioengineering strategies provide promising avenues to overcome several of these challenges. Surface modifications to enhance brain targeting, optimization of cargo loading strategies tailored to disease‐specific pathology, and approaches to improve circulation stability and biodistribution are actively being explored [222]. Nonetheless, engineered sEVs may still accumulate in off‐target organs, such as the liver and spleen, prompting investigation into advanced targeting solutions, including magnetic guidance of magnetite‐loaded vesicles or stimulus‐responsive release systems. Importantly, despite these limitations, accumulating preclinical evidence supports the capacity of bioengineered neural sEVs to enhance therapeutic efficacy in AD and PD models by delivering appropriate molecular cargo to defined cellular targets [17, 22].

Looking ahead, the successful clinical translation of sEV‐based therapies will require coordinated efforts to establish standardized protocols for isolation, characterization, loading, storage, and quality control, alongside robust biodistribution and pharmacokinetic studies. Expansion of existing frameworks such as MISEV, and potentially integrating principles from MIRIBEL and ARRIVE guidelines, could further strengthen experimental harmonization, enable future meta‐analyses and AI/ML‐driven integrative approaches, and improve regulatory alignment [134]. Equally important will be close collaboration among academia, industry, and regulatory authorities to align technological advances with regulatory expectations. Collectively, the convergence of bioengineering innovations with sEV biology offers a compelling and realistic pathway toward translating the inherent advantages of neural cell‐derived sEVs, such as biocompatibility, BBB permeability, and cell‐specific tropism, into clinically viable therapies for NDDs.

## 8 Conclusions and Perspectives

Collectively, the evidence reviewed herein positions sEVs as highly versatile and biologically attuned platforms for therapeutic intervention in NDDs. Their endogenous origin confers key advantages over synthetic nanocarriers, including superior biocompatibility, low immunogenicity, prolonged stability in circulation, and an inherent capacity for intercellular communication. Importantly, sEVs can access the CNS via multiple administration routes and traverse restrictive brain barriers, making them particularly attractive candidates for addressing the long‐standing challenges associated with CNS drug delivery.

While mesenchymal stem cell–derived sEVs have dominated early translational efforts, this review highlights a growing body of evidence demonstrating that neural cell–derived sEVs, including those from neural stem cells, astrocytes, microglia, neurons, and brain endothelial cells, possess distinct molecular signatures, organotropic properties, and functional cargos that may be better suited for targeting CNS pathology. Notably, sEVs derived from neural stem cells and region‐specific glial populations often outperform non‐neural sEVs in terms of neuroprotection, synaptic support, mitochondrial rescue, and modulation of neuroinflammation. These findings underscore neural sEVs as an underexplored yet highly promising class of biologically specialized vesicles for both disease modification and precision delivery applications in NDDs.

Beyond their intrinsic therapeutic effects, neural sEVs offer substantial flexibility for bioengineering. Advances in donor cell modification, surface functionalization, and endogenous or exogenous cargo‐loading strategies have enabled the delivery of diverse therapeutic modalities, including small molecules, proteins, mRNAs, and regulatory miRNAs, with improved targeting specificity. Such approaches provide opportunities to overcome critical barriers such as limited BBB permeability, off‐target exposure, and cargo instability. Importantly, several studies now demonstrate that engineered neural sEVs can achieve meaningful functional outcomes in relevant in vivo models, supporting their potential as next‐generation, multifunctional nanotherapeutics.

Despite these encouraging advances, significant challenges remain before neural sEV‐based therapies can be translated into clinical practice. Key unresolved issues include incomplete understanding of sEV biodistribution, pharmacokinetics, and long‐term safety, as well as limited mechanistic insight into how distinct sEV subpopulations traverse brain barriers and interact with specific CNS cell types. Moreover, therapeutic outcomes are highly dependent on donor cell source, cellular state, and sEV cargo composition, emphasizing the need for systematic molecular characterization and rigorous comparative studies. Equally critical are challenges related to scalability, batch‐to‐batch reproducibility, cargo loading efficiency, storage stability, and the lack of standardized isolation and characterization protocols across studies.

Looking ahead, progress in this field will require coordinated efforts to integrate mechanistic biology, advanced bioengineering, and standardized methodological frameworks, in line with evolving community guidelines. A deeper understanding of neural sEV heterogeneity, context‐dependent functionality, and cell‐specific targeting will be essential for rational therapeutic design. Ultimately, the convergence of these advances holds the potential to establish neural cell–derived sEVs as clinically viable, precision therapeutics for CNS disorders, either as standalone biologics or as highly adaptable delivery vehicles capable of addressing the complex, multifactorial nature of neurodegenerative diseases.

## Author Contributions

The manuscript is jointly written and edited by M.W.S., W.Z., L.C.P., Y.W., and A.C. The figures are prepared by M.W.S., while the overall planning of the manuscript is coordinated by M.W.S., Y.W., and A.C. All authors contribute equally to the critical review of the final version, including the figures.

## Conflicts of Interest

The authors declare no conflicts of interest.

## Data Availability

The authors have nothing to report.
